# Effectiveness and Cost-effectiveness of an Empowerment-Based Self-care Education Program on Health Outcomes Among Patients With Heart Failure: A Randomized Clinical Trial

**DOI:** 10.1001/jamanetworkopen.2022.5982

**Published:** 2022-04-05

**Authors:** Doris Sau-fung Yu, Polly Wai-chi Li, Shirley Xue Li, Robert D. Smith, Sunny Chiu-Sun Yue, Bryan P. Y. Yan

**Affiliations:** 1 School of Nursing, Li Ka Shing Faculty of Medicine, The University of Hong Kong, Hong Kong; 2 Department of Pharmacology and Pharmacy, Li Ka Shing Faculty of Medicine, The University of Hong Kong, Hong Kong; 3 Department of Medicine and Geriatrics, United Christian Hospital, Hong Kong; 4 Division of Cardiology, Department of Medicine and Therapeutics, Faculty of Medicine, The Chinese University of Hong Kong, Hong Kong

## Abstract

**Importance:**

Strategies that enhance self-care of patients with heart failure reduce mortality and health care use.

**Objective:**

To examine whether an empowerment-based self-care education program was more effective and cost-effective to improve self-care, health status, and hospital service use than a didactic education program in patients with heart failure.

**Design, Setting, and Participants:**

In this double-blind randomized clinical trial, a consecutive sample of 988 patients with heart failure from the cardiac clinics of 2 regional hospitals underwent eligibility screening from February 1, 2017, to May 31, 2019, using the criteria of age of 55 years or older, heart failure diagnosed 6 months before screening, and New York Heart Association class II to IV. A total of 236 participants were randomized to the empowerment (n = 118) or education (n = 118) group.

**Interventions:**

The 12-week, group-based, empowerment-based education program included self-care assessment, goal-orientated actions in symptom recognition and response, fluid and dietary modification, and lifestyle management. Didactic education covered the same topics without empowerment strategies.

**Main Outcomes and Measures:**

The primary outcome was self-care measured by the Self-care Heart Failure Index (SCHFI) maintenance, management, and symptom perception subscales at posttest and 3-month end points. Secondary outcomes included measures of knowledge, confidence, health-related quality of life, and health service use. Direct, indirect, and social costs of the 2 programs were collected for cost-effectiveness analysis.

**Results:**

A total of 236 Chinese patients (mean [SD] age, 70 [8.0] years; 149 [63.1%] men) were included in the study. The empowerment group reported significantly greater improvement in SCHFI management scores (mean difference, 13.76; 95% CI, 5.89-21.62; Cohen *d* = 0.46 at posttest and Cohen *d* = 0.35 at 3 months) and symptom perception scores (mean difference, 20.36; 95% CI, 13.98-26.75; Cohen *d* = 0.84 at posttest and Cohen *d* = 0.61 at 3 months). The empowerment group had lower risks of emergency department attendance (incidence rate ratio, 0.55; 95% CI, 0.31-0.95; *P* = .03) and hospital admission (incidence rate ratio, 0.38; 95% CI, 0.21-0.68; *P* = .001) and better improved self-care knowledge (change in score [empowerment minus education], 1.29; 95% CI, 0.48-2.09) and confidence (change in score [empowerment minus education], 7.98; 95% CI, 1.91-14.05). Empowerment was cost-saving and cost-effective at T2 compared with the education group for quality-adjusted life-years, with an incremental cost-effectiveness ratio of −114 485.

**Conclusion and Relevance:**

In this randomized clinical trial, an empowerment approach led to clinically relevant improvement in symptom perception and self-care management among patients with heart failure. Its cost-effectiveness in improving these prognostic factors also benefits the patient-reported outcome.

**Trial Registration:**

Chinese Clinical Trial Registry: ChiCTR-IOR-16008254

## Key Points

**Question:**

What are the effectiveness and cost-effectiveness of a 12-week, empowerment-based self-care intervention on health outcomes and health care use among patients with heart failure?

**Findings:**

In this randomized clinical trial of 236 patients with heart failure, when compared with didactic education, an empowerment-based self-care intervention was effective and cost-effective, resulting in improvement in symptom perception and response.

**Meaning:**

These findings suggest that education using an empowerment approach is more effective than didactic methods to improve self-care and health outcomes of patients with heart failure.

## Teaser

This randomized clinical trial compares self-care and cost-effectiveness outcomes between patients with heart failure who received an empowerment-based self-care program vs those who received a didactic educational program.

## Introduction

Heart failure has evolved as a global epidemic, affecting more than 64 million people worldwide, with a cost of illness of US $346.17 billion.^1^ Although most spending is used for hospitalization for exacerbated heart failure,^2^ approximately 40% of cases are preventable by effective self-care.^3^ Heart failure–related self-care refers to a dynamic cognitive-behavioral process aimed at maintaining health through adherence to pharmacological and nonpharmacological treatment (self-care maintenance), proactive symptom monitoring (symptom perception), and the accurate interpretation of and response to early cues of disease deterioration (self-care management).^4^ Because increasing research evidence indicates the role of effective self-care in determining patient-reported health outcomes,^5,6^ more recent international heart failure management guidelines advocate for self-care as a crucial treatment component to complement pharmacological care.^4^

Although numerous trials have addressed this advocacy by testing different self-care enhancement models, science implementation was hindered by the large variations in model design, inconsistent research findings,^7^ and the resulting failure to identify crucial program characteristics related to clinical outcomes.^8^ More recent evidence indicates that effective symptom recognition (ie, symptom perception) together with appropriate response (ie, self-care management) are crucial to prevent further cardiac decompensation and delayed care seeking,^9,10^^,^ thereby reducing mortality and hospital service use.^11,12^ However, the published self-care enhancement models have seldom integrated explicit methods to strengthen the complex cognitive process of symptom perception and response.^10^ Even less was done to examine the economic benefit of the self-care model in making clinically meaningful change in behavior and clinical outcomes. This study aimed to (1) compare the effects of an empowerment-based self-care program and a didactic educational program on self-care domains (primary outcome), including self-care maintenance, management and symptom perception, self-care knowledge and self-efficacy, health-related quality of life (HRQL), and emergency department (ED) attendance and hospital admission of patients with heart failure; and (2) investigate whether an empowerment approach is more cost-effective for improving self-care and HRQL and reducing health care service use.

## Methods

### Study Design and Participants

This double-blind randomized clinical trial recruited outpatients from the cardiac units of 2 regional hospitals that served 2 geographic districts with the highest population density^13^ in Hong Kong. All participants provided written informed consent. The study was approved by the Hospital Authority Clinical Research Ethics Committee. The study followed the Consolidated Standards of Reporting Trials (CONSORT) reporting guideline for randomized clinical trials.^14^ The trial protocol is available in Supplement 1.

Participant recruitment occurred from February 1, 2017, to May 31, 2019. The inclusion criteria were (1) community-dwelling and age of 55 years or older, (2) heart failure diagnosed 6 months before screening, and (3) New York Heart Association (NYHA) grade of class II to IV. The last 2 criteria excluded those who may not perform self-care because they lacked illness and symptom experience. The exclusion criterion was inability to participate because of impaired communication and cognition.

Participants gave written informed consent before the nurse conducted an in-person baseline outcome evaluation in the clinics. Randomization with permuted blocks of 8, 10, or 12 using sequentially numbered, opaque, sealed envelopes was used to allocate participants to receive the empowerment-based self-care program or didactic education in a 1:1 ratio. The participants had no information about which program was the test intervention.

For sample size estimation, referring to the effect size reported for the self-care enhancement program on self-care and HRQL (Cohen *d* = 0.45-1.24),^15,16,17^ 118 individuals per arm were needed to detect small to moderate effects (Cohen *d* = 0.4) of the empowerment-based programs on primary and secondary outcomes at 80% power and a 5% level of significance, assuming an autocorrelation of 0.4 within the intracorrelated outcomes and a 20% attrition rate.^18^ Posttest outcome evaluation was conducted on completion of the 12-week intervention and at 3 months thereafter by a research assistant blinded to group allocation.

### Empowerment-Based Self-care Program

The 12-week, empowerment-based self-care program was underpinned by empowerment theory^19^ and contained educational content that complied with international heart failure clinical guidelines. The key program focuses were to empower patients to develop personalized goals for heart failure–related self-care improvement and use patient-professional partnerships to optimize their skills, resource availability, and support for goal attainment. Led by a cardiac nurse, the program included 5 face-to-face group sessions (4-5 patients per group) on guideline-based topics, including (1) heart failure manifestations and symptom monitoring, (2) dietary and fluid modification, (3) medication management, (4) deteriorating symptom recognition and management, and (5) advice on remaining physically active. The group sessions were followed by 3 weekly and then biweekly telephone calls.

The incorporation of a goal-setting process in each face-to-face session was central to the empowerment-based design.^20^ The nurse first invited the patients to describe their self-care practice for the corresponding topic, which was followed by structured education by the nurse on desirable self-care practices. The purpose of this discussion was to create a contrast so that the participants realized their own self-care deficits and the associated negative health consequence. Then the nurse motivated and supported participants to set goals and action plans for self-care improvement. The nurse used several strategies to empower goal attainment: (1) provided training on tactical and situational skills related to self-care, such as monitoring symptoms, managing fluids and medication, maintaining an active lifestyle, and seeking medical care promptly; (2) optimized peer influence; and (3) addressed the patients’ anticipated barriers and challenges in goal attainment through health counseling. In the subsequent follow-up telephone calls, the nurse monitored the progress of goal attainment and gave further health counseling to address the barriers to self-care if indicated.

### Didactic Education

The 12-week didactic educational program was delivered by another cardiac nurse. The program comprised 5 weekly face-to-face sessions (45 minutes each) to deliver didactic education on the same topics as the empowerment-based self-care program, followed by 3 follow-up telephone calls over 7 weeks to reinforce the relevant health advice. No empowerment strategies were included.

### Outcome Measures

All the outcome measures except health service use were measured by validated measures (Chinese versions) in face-to-face interviews at baseline and through telephone interviews at the posttest and 3-month time points. The primary outcomes were measured using the 29-item Self-Care Heart Failure Index (SCHFI), version 7.2, which comprises 3 subscales to measure self-care maintenance, self-care monitoring, and symptom perception. The standardized subscale scores range from 0 to 100, with a score of 70 or higher indicating adequate self-care^21,22^ and an 8-point difference indicating a minimal clinically important difference (MCID).^22^ The Cronbach α values were 0.71 for self-care maintenance, 0.68 for management, and 0.74 for symptom perception in this study.

The secondary outcomes included self-care knowledge and self-efficacy, HRQL, and health service use. The 15-item Dutch Heart Failure Knowledge Scale^23^ and the 10-item Self-care Self-efficacy Scale^24^ scores captured self-care knowledge and self-efficacy, respectively, with higher scores reflecting stronger attributes. The Cronbach α values were 0.72 for the Dutch Heart Failure Knowledge Scale and 0.87 for the Self-care Self-efficacy Scale in this study, and the Self-care Self-efficacy Scale was cross-culturally valid when used in the Hong Kong Chinese population.^24^ The 21-item Minnesota Living with Heart Failure Questionnaire (MLHFQ) measured HRQL, with a higher score indicating poorer HRQL.^25^ A mapping algorithm^26^ converted the MLHFQ score to the 5-level EuroQoL-5D utility score (ie, estimated quality-adjusted life-year [QALY]) for the economic evaluation of the empowerment-based self-care program. Health service use in terms of cardiac-related ED attendance and cardiac-related hospital admission was retrieved from electronic hospital records for a period of up to 6 months after the baseline assessment. Information about the index diagnosis, the nature of the admission venue, and the length of stay was recorded.

### Statistical Analysis

The baseline equivalence of the 2 study arms was examined by the χ^2^ and 2-tailed, independent *t* tests. A generalized estimating equation (GEE) model was used to examine the effects of the empowerment-based self-care program on patient-reported outcomes based on the intention-to-treat principle.^27^ The interaction term of time × group was included in the GEE model to compare changes in the self-care–related outcomes, self-care knowledge, self-efficacy, and HRQL between the 2 study groups across the baseline and posttest 3-month evaluation period, with adjustment for potential confounding variables as appropriate (ie, baseline group differences at 2-sided *P* < .25). The mean difference in the changes of outcomes from baseline to the 2 posttest end points were compared between the study groups, and the effect sizes (Cohen *d*) were computed.

A negative binomial regression was used to identify between-group differences in ED attendance and the number of hospital admissions because these data may be overdispersed in the sample. Cox proportional hazards regression analysis was performed to compare the between-group difference in the time to event, and the odd ratios for these hospital service use outcomes were computed. Statistical analyses were performed using SPSS software, version 25 (IBM Corp), with α = .05.

Cost analysis was performed by comparing the between-group cost difference (including intervention, health service, and societal cost) in improving the SCHFI domains and MLHFQ using incremental cost-effectiveness ratios (ICERs) over an approximately 6-month analytic time horizon (from baseline to 3 months posttest). Cost-effectiveness analysis was performed by using the decision tree analytical models with Markov cohort simulation. With the education group used as the reference, the cost-effectiveness of the empowerment-based self-care program was evaluated by computing the incremental cost per unit of (1) MCID in self-care domains of SCHFI and (2) estimated QALYs gained. A bias-corrected accelerated bootstrapping method computed the 95% CI of the ICERs, and cost-effectiveness acceptance curves were generated for all cost-effectiveness measurements. The effects of uncertainties from the model parameters were evaluated by deterministic and probabilistic sensitivity analyses using 10 000 Monte Carlo iterations. Following the World Health Organization’s guidelines,^28^ we defined the willingness-to-pay (WTP) threshold as 1 times the local gross domestic product per capita (US $46 081).^29^ Interventions with an ICER below the threshold were considered cost-effective. Further details of the economic evaluation, including utility, cost estimation, health status, and ICER, are provided in the eMethods, eTable 1 (parameters for the cost estimation), and eTable 2 (distributions for transitional probability, costs, and utility) in Supplement 2.

Statistical analyses on cost-effective analysis were performed using R (R Foundation for Statistical Computing), TreeAge Pro Healthcare (TreeAge Software), and heRo3 (GoPro Inc). All other statistical analyses were performed using SPSS software, version 25 (SPSS Inc), with α = .05.

## Results

### Study Participants

**Figure 1.**
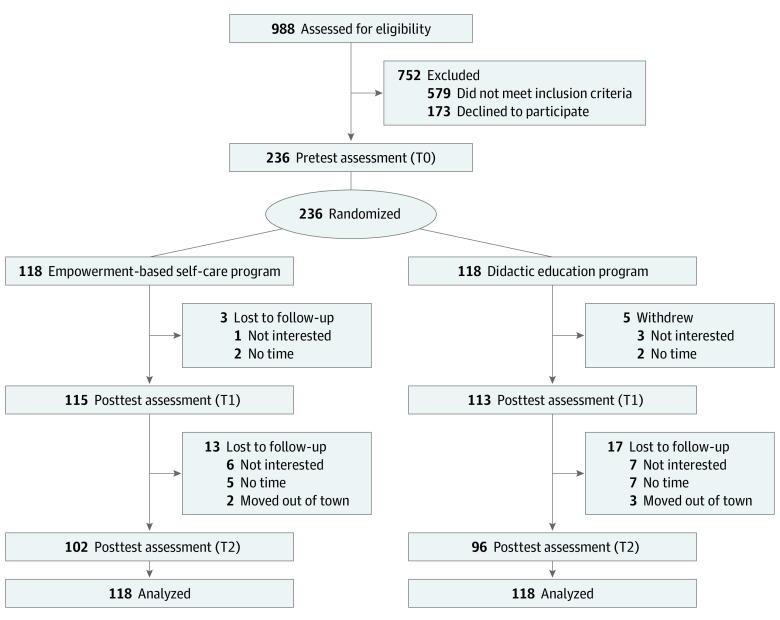
Flow Diagram of Study Participation T0 indicates baseline; T1, postintervention; and T2, 3-month postintervention.

Of the 988 patients who consecutively attended the clinics, 236 Chinese patients (mean [SD] age, 70 [8.0] years; 149 men [63.1%] and 87 women [36.9%]) were evaluated (Table 1). A total of 191 patients (80.9%) had NYHA class II HF. The baseline SCHFI scores indicated their suboptimal self-care maintenance, management, and symptom perception (ie, standardized scores <70). No between-group differences were found in the baseline characteristics. A total of 198 patients completed the 3-month posttest assessment, with 102 (86.4%) from the empowerment group and 96 (81.4%) from the education group (Figure 1).

**Table 1.** Baseline Characteristics of the 236 Study Participants^a^

Characteristic	Empowerment group (n = 118)	Education group (n = 118)
Sociodemographic characteristics		
Age, mean (SD), y	69.1 (7.7)	70.7 (8.3)
Sex		
Male	79 (66.9)	70 (59.3)
Female	39 (33.1)	48 (40.7)
Educational level		
Primary or below	58 (49.6)	64 (54.2)
Secondary	39 (33.3)	42 (35.6)
Tertiary or above	20 (17.1)	12 (10.2)
Marital status		
Single, divorced, or widowed	25 (21.2)	30 (25.4)
Married	93 (78.8)	88 (74.6)
Living alone		
No	101 (87.1)	103 (87.3)
Yes	15 (12.9)	15 (12.7)
Clinical characteristics		
Time since diagnosis of chronic heart failure, median (IQR), mo	29.5 (14.0-56.0)	32.5 (16.0-62.0)
NYHA class		
II	96 (81.4)	95 (80.5)
III	22 (18.6)	23 (19.5)
Cause of heart failure		
Coronary artery disease	59 (50.0)	54 (45.8)
Dilated cardiomyopathy	25 (21.2)	23 (19.7)
Valvular heart disease	30 (25.4)	26 (22.2)
Atrial fibrillation	48 (40.7)	45 (38.5)
Hypertension	63 (53.4)	69 (58.5)
No. of hospitalizations in the last year		
0	44 (37.3)	50 (42.4)
1	35 (29.7)	32 (27.1)
2-3	23 (19.5)	27 (22.9)
≥4	16 (13.6)	9 (7.6)
LVEF, mean (SD), %	43.0 (14.2)	44.0 (13.7)
Hemoglobin, mean (SD), g/dL	13.2 (1.7)	13.0 (1.7)
Blood urea nitrogen, mean (SD), mg/dL	26.61 (28.85)	25.77 (28.01)
Serum creatinine, mean (SD), mg/dL	1.17 (0.46)	1.18 (0.64)
Charlson comorbidity index, mean (SD)	2.2 (1.2)	2.3 (1.3)
Outcome scores, mean (SD)		
Self-care Heart Failure Index		
Maintenance	59.11 (16.56)	55.33 (14.91)
Management	55.87 (19.57)	54.38 (21.93)
Symptom perception	58.77 (17.17)	55.13 (17.34)
Confidence	72.35 (19.13)	70.19 (19.72)
Dutch Heart Failure Knowledge Scale	8.13 (2.05)	7.43 (2.53)
Minnesota Living with Heart Failure Questionnaire	33.91 (18.69)	31.07 (17.74)

Abbreviations: LVEF, left ventricular ejection fraction; NYHA, New York Heart Association.

SI conversion factors: To convert hemoglobin to grams per liter, multiply by 10; urea nitrogen to millimoles per liter, multiply by 0.357; creatinine to micromoles per liter, multiply by 88.4.

^a^ Data are presented as number (percentage) of patients unless otherwise indicated.

### Primary Outcomes

The empowerment group reported more positive changes than the education group for self-care management and symptom perception at both the immediate and 3-month posttest end points, with effect size ranges of 0.35 to 0.46 for self-care management and 0.61 to 0.84 for symptom perception (Table 2). The mean difference between groups was beyond the criterion level for an MCID, showing a clinically important benefit for the intervention. The GEE results indicated significant time × group interaction effects for these outcomes that favored the empowerment group.

**Table 2.** Effects of the Empowerment-Based Self-care Program

Patient-reported outcome	Mean (SD)		Treatment effect (95% CI)		Time × group interaction effects by GEE	
	Empowerment group	Education group	Difference in change of score from baseline (empowerment − education)	Effect size (Cohen *d*)	B (95% CI)	*P* value
**SCHFI maintenance**						
Baseline	59.11 (16.56)	55.33 (14.91)	NA	NA	NA	NA
Posttest	71.01 (14.48)	66.90 (14.43)	1.89 (−2.81 to 6.60)	0.10	2.19 (2.34 to −2.41)	.35
3-mo posttest	80.77 (16.38)	78.37 (16.34)	−1.39 (−6.98 to 4.21)	−0.069	−3.28 (−5.71 to 5.05)	.91
**SCHFI management**						
Baseline	55.87 (19.57)	54.38 (21.93)	NA	NA	NA	NA
Posttest	67.18 (23.01)	52.50 (22.30)	13.76 (5.89 to 21.62)	0.46	13.77 (6.07 to 21.46)	<.001
3-mo posttest	58.84 (22.76)	47.53 (20.73)	10.11 (1.96 to 18.25)	0.35	10.98 (3.21 to 18.75)	.006
**SCHFI symptom perception**						
Baseline	58.77 (17.17)	55.13 (17.17)	NA	NA	NA	NA
Posttest	82.32 (16.18)	57.94 (22.94)	20.36 (13.98 to 26.75)	0.84	17.34 (10.91 to 23.76)	<.001
3-mo posttest	71.41 (18.80)	54.76 (21.60)	12.47 (6.02 to 19.92)	0.61	9.36 (2.85 to 15.87)	.005
**SCSES self-care confidence**						
Baseline	72.35 (19.13)	70.19 (19.72)	NA	NA	NA	NA
Posttest	80.54 (18.71)	69.37 (21.92)	7.98 (1.91 to 14.05)	0.35	7.80 (1.83 to 13.77)	.01
3-mo posttest	74.08 (21.42)	68.74 (19.95)	3.77 (−3.09 to 10.64)	0.15	1.74 (−4.78 to 8.25)	.61
**DHFKS**						
Baseline	8.13 (2.05)	7.43 (2.53)	NA	NA	NA	NA
Posttest	9.97 (2.74)	7.99 (3.50)	1.29 (0.48 to 2.09)	0.418	1.29 (0.49 to 2.09)	.002
3-mo posttest	8.99 (4.14)	7.31 (4.16)	0.98 (−0.10 to 2.07)	0.23	0.98 (−0.93 to 2.06)	.07
**MLHFS**						
Baseline	33.91 (18.69)	31.07 (17.74)	NA	NA	NA	NA
Posttest	18.77 (13.31)	23.68 (18.27)	−6.65 (−10.78 to −2.53)	0.43	−6.61 (−10.68 to −2.55)	.001
3-mo posttest	20.21 (17.23)	22.57 (19.00)	−5.08 (−9.57 to −0.60)	0.34	−3.65 (−8.12 to 0.89)	.11
**Estimated QALYs at 1 y**						
Baseline	0.68 (0.14)	0.69 (0.13)	NA	NA	NA	NA
Posttest	0.80 (0.10)	0.75 (0.13)	0.05 (0.02 to 0.08)	0.43	0.05 (0.02 to 0.08)	.001
3-mo posttest	0.78 (0.12)	0.77 (0.14)	0.04 (0.004 to 0.07)	0.32	0.03 (−0.006 to 0.06	.12

Abbreviations: DHFKS, Dutch Heart Failure Knowledge Scale; GEE, generalized estimating equation; MLHFS, Minnesota Living with Heart Failure Scale; NA, not applicable; QALY, quality-adjusted life-year; SCHFI, Self-care Heart Failure Index; SCSES, Self-care Self-efficacy scale.

### Secondary Outcomes

The empowerment group reported greater improvement than the education group in self-care knowledge, self-efficacy, and HRQL at the first time point, with effect sizes ranging from 0.35 to 0.43 (Table 2). The GEE model also identified time × group interaction effects at this time point but not at 3 months thereafter.

**Figure 2.**
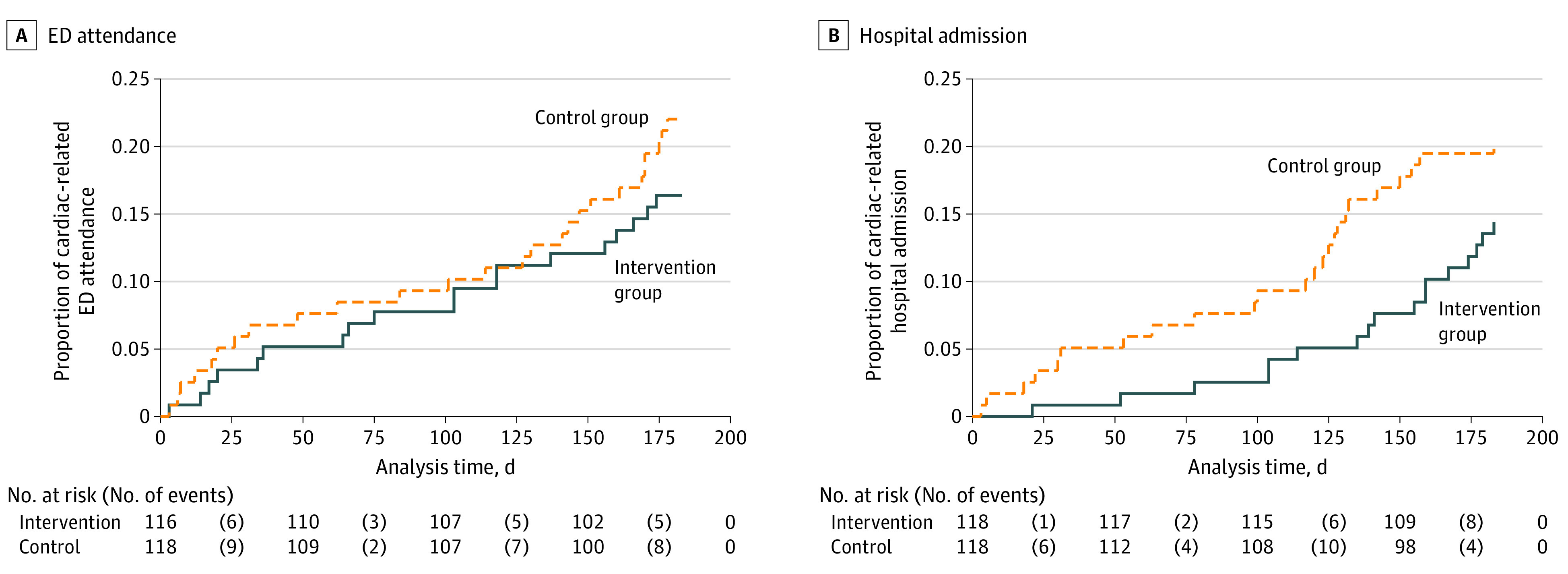
Kaplan-Meier Curves of Health Service Use Between the Intervention and Control Groups ED indicates emergency department.

The number of cardiac-related ED attendances and hospital admissions was lower in the empowerment group than the education group, with the incidence rate ratio (IRR) indicating that the reduction was statistically significant (empowerment vs control: cardiac-related ED attendance = 24 vs 44; IRR, 0.55; 95% CI, 0.31-0.95; *P* = .03; cardiac-related hospital admission = 18 vs 48; IRR, 0.38; 95% CI, 0.21-0.68; *P* = .001). However, although the time to cardiac-related ED attendance and cardiac-related hospital admission was longer for the empowerment group than the education group, the hazard ratio did not show significant difference (cardiac-related ER attendance: hazard ratio, 0.74; 95% CI, 0.39-1.29; cardiac-related hospital admission: hazard ratio, 0.65; 95% CI, 0.34-1.22) (Figure 2A and B).

### Cost Analysis

**Figure 3.**
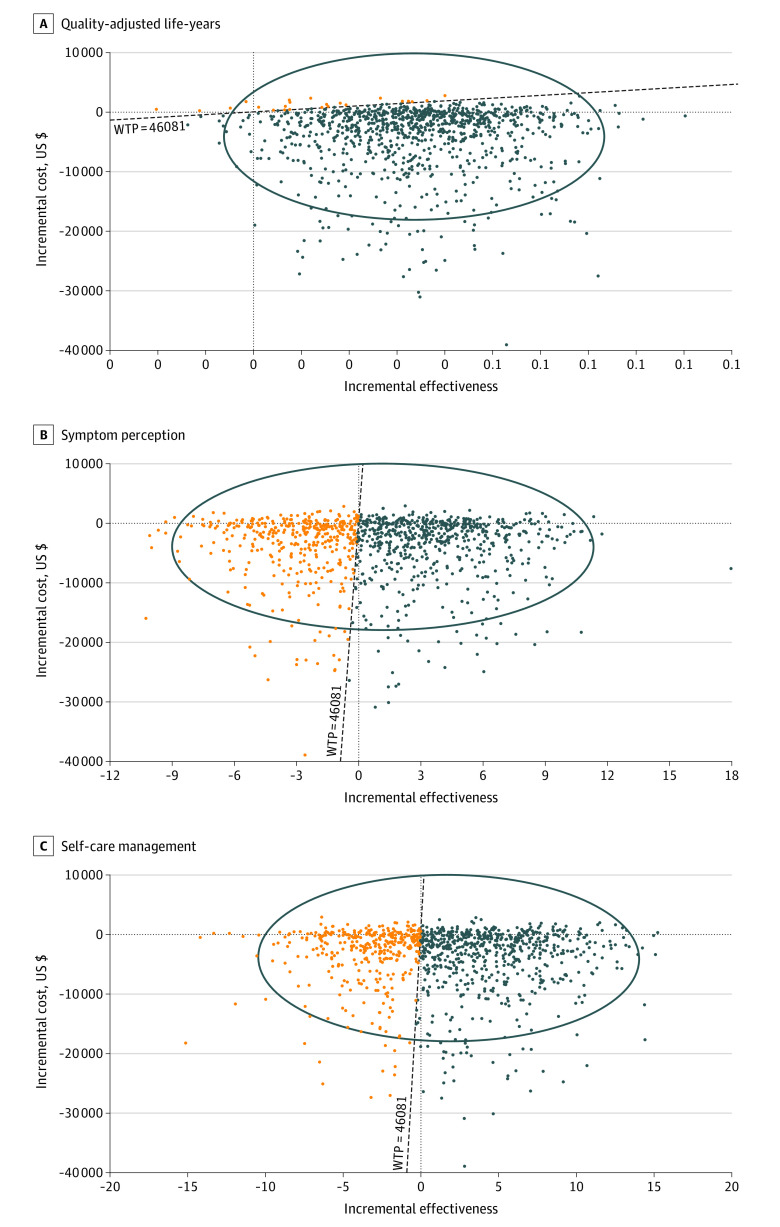
Cost-effectiveness Plane for Empowerment vs Education Blue dots represent simulations showing the empowerment-based self-care program as cost-saving; orange dots, simulations showing the program as not cost-saving. WTP indicates willingness to pay.

The total cost was significantly greater for the education group than the empowerment group during the 6-month outcome evaluation period (empowerment vs education = US $461.9 vs $834.0, *P* = .005) because of the higher hospital admission rate of the education group. With US $46 081 (Hong Kong gross domestic product per capita in 2017) used as the WTP threshold, the empowerment-based self-care program was cost-saving compared with the educational program and was below the WTP threshold. Among the 10 000 iterations included in the simulation, the ICER for the increase in the number of QALYs attributable to the empowerment intervention was −114 485. The empowerment-based self-care program was also more cost-effective for producing MCIDs for the SCHFI symptom perception (ICER = −2946) and management (ICER = −2533). The probabilistic analysis of QALYs, SCHFI symptom perception, and SCHFI management showed that the empowerment intervention, compared with education, had higher probability of cost-effectiveness under the WTP threshold in 98% of QALY simulations, 83% of SCHFI symptom perception simulations, and 83% of SCHFI management simulations (Figure 3). Acceptability curves showed higher probability of cost-effectiveness for empowerment across ranges of WTP thresholds for QALYS, symptom perception, and management (eFigures 1-3 in Supplement 2). The probabilistic analysis showed greater uncertainty in cost-effectiveness for the SCHFI domains (83% of simulations under the WTP threshold for both symptom perception and management) compared with the cost-effectiveness of decreasing the number of QALYs.

## Discussion

Self-care is an important prognostic indicator of HF-associated morbidity and mortality.^30,31^ To our knowledge, this is the first randomized clinical trial to evaluate the superior effects of an empowerment-based self-care program, compared with didactic education, for improving the symptom perception, self-care management, and HRQL of patients with heart failure at a clinically significant level. The approach was cost-saving and cost-effective for improving both self-care and QALY outcomes.

As more research points to the roles of effective symptom perception and self-care management in preventing avoidable cardiac decompensation,^9,32,33,34^ this study supports the benefit of using an empowerment approach to enhance these self-care practices. In fact, a systematic review^35^ identified the challenges of engaging patients in complex decision-making of accurate symptom recognition, interpretation (ie, symptom perception), and responses to promptly reverse early disease deterioration (ie, self-care management). The critical features of the empowerment approach in leading to such changes may include the deliberate use of tailoring care in goal setting to meet the patients’ literacy, resource and capacity, and self-care needs. The use of explicit methods, such as data plotting for body weight and critical symptoms, increased patients’ awareness of bodily changes. Together with cues to action, the decision-making process in symptom perception and response was explicated. Systematic reviews^9,36^ on heart failure–related self-care intervention have also suggested the potential of these design features in optimizing clinical and health outcomes. Indeed, the more positive findings reported in this study may be related to typical cultural orientations, including face-saving and maintaining social integrity among Chinese older adults, which drove them to have better self-care goal attainment.^37,38^ Their exaggerated power relationship with health care professionals, which originated from the professional status and life-saving ability of physicians and nurses, may inspire them to regard adherence to self-care instruction as an obligation.^38,39^

However, our study failed to identify any benefits of the empowerment approach in terms of shortening the time to the first ED attendance or hospital admission. This result is contradictory to a previous study^40^ that reported a positive effect of a nurse-led self-care clinic for delaying the time to health care service use within 12 months. The shorter evaluation timeframe and the more stable clinical condition of our study participants (80% with NYHA class II) may explain the nonsignificant findings. An alternative explanation may be related to the increased care-seeking ability of the empowerment group during early disease deterioration. The lower cost on hospital service consumption for this group may suggest that their prompt action has prevented more serious medical events.

The results of our economic evaluation were encouraging; the empowerment-based program was cost-saving and cost-effective compared with the more conventional educational approach in making clinically significant changes in self-care and HRQL. Even though the empowerment-based model incurred more nursing time cost, this was outweighed in the negative ICER value, which reflected the economic benefit of the empowerment program that resulted from a reduction in health care service spending associated with hospital admissions compared with the education approach. These results concur with previous studies^41,42^ that have identified ineffective HF-related self-care as a significant prognostic indicator of avoidable morbidity and mortality risk.

### Limitations

This study has some limitations. First, the sample was overrepresented by patients with NYHA II and III heart failure because those with more severe clinical presentation found it difficult to attend the in-person empowerment sessions. The external validity was further threatened by recruiting participants from the cardiac clinics of 2 regional hospitals in Hong Kong, which only covered approximately 11% of the hospital beds in the public sector. Second, although the training schedule was similar between the study groups in terms of the number of in-person and telecare sessions, there was more intensive nurse-patient interaction during the goal attainment process, and varied levels of attention may be a confounder in the outcome evaluation. Indeed, the lack of a no-treatment control group and a longer-term evaluation meant that we could not identify a sustained benefit of the empowerment program over standard care. Third, medical costs were estimated based on the generic expense for 1 ED attendance and hospital admission, as published by the Hong Kong Hospital Authority, rather than the actual cost of the medical services used by the patients.

## Conclusions

The empowerment-based self-care program developed in this randomized clinical study was more cost-effective than the conventional educational approach for providing clinically significant improvements in symptom perception, self-care management, and HRQL in patients with heart failure. The positive effect on hospital service use also supports the adoption of this care model in the health care context.

## Notes

Supplement 1.
Trial Protocol

Supplement 2. **eMethods.** Description of Economic Evaluation **eTable 1.** Parameters of Intervention Cost, Direct Medical and Social Cost for Cost-effectiveness Analysis **eTable 2.** Parameters Definition for Cost-effectiveness Model **eFigure 1.** Acceptance Curve of Empowerment and Education Control – Quality-Adjusted Life-year **eFigure 2.** Acceptance Curve of Empowerment and Education Control – Symptom Perception **eFigure 3.** Acceptance Curve of Empowerment and Education Control – SCHFI Management

Supplement 3.
Data Sharing Statement
