# Supplementary material for: Effectiveness and Cost-effectiveness of an Empowerment-Based Self-care Education Program on Health Outcomes Among Patients With Heart Failure: A Randomized Clinical Trial
Source: JAMA Netw Open. 2022 Apr 5;5(4):e225982. doi: 10.1001/jamanetworkopen.2022.5982 (PMC8984788; doi:10.1001/jamanetworkopen.2022.5982)

<u>**Trial Protocol**</u>

Effectiveness and cost-effectiveness of an Empowerment-based self-care education program on health outcomes in Patients with heart failure: A Randomized Trial

Prof. Doris Yu

Table of Contents

**Background information**	**p.2-3**
**Research Program**	
**Purpose of the study**	**P.3**
**Hypotheses**	**P.3**
**Study population**	**P.3-4**
**Study intervention**	**P.4-6**
**Study outcomes**	**P.6**
**Statistical analysis**	**P.6-7**
**Ethical considerations**	**P.7**
**Impact and Future Direction**	**P.7-8**
**References**	**P.9-10**
**Additional Materials**	
**1. User Manual of the empowerment-based self-care education program**	**P.11-19**
**2. Self-monitoring Form**	**P.20-22**

Background Information

Heart failure (HF) is a complex clinical syndrome resulting from inefficient myocardial pumping, and is commonly presented as the end manifestation of many cardiac diseases. Similar to other organ failure, HF is characterised by a typical progressively deteriorating trajectory punctuated by serious episodes of acute disease decompensation.[1] The burden of HF is, therefore, not only manifested in distressing symptoms, functional disability and compromised quality of life, but also in frequent hospital admission. Recent Hong Kong data reported that HF was associated with more than 19,000 hospital admissions per annum (i.e., 53 admissions per day) and an unplanned re-admission rate within 4 weeks of up to 25%.[2] However, there is convincing evidence to indicate that around 40% of hospital admissions could be avoided if patients demonstrate consistent self-care.[3-4].

Self-care is defined as a naturalistic decision-making process that includes behaviors to maintain physiological stability (self-care maintenance) and respond to changing disease conditions (self-care management).[5] The critical self-care of HF includes self-monitoring of body weight and peripheral swelling to detect fluid overload, adhering to medications, restricting dietary sodium and fluid intake, and proactively responding to, untoward signs and symptoms of disease deterioration. Despite more than a decade of research into self-care strategies, self-care in the CHF population remains very poor.[6] A study led by the project team revealed that more than 90% (n=256) of the community-dwelling HF patients did not monitor their body weight, more than 35% did not observe for peripheral edema. Such poor self-monitoring behaviors explained why no patients had adjusted the dose of diuretic according to their fluid status when flexible diuretic regime was prescribed.[7] Moreover, approximately 22 - 40% seldom followed the dietary advice. More than a quarter of the patients could not rapidly recognize symptom exacerbation and around 70% of them did not attempt to take any self-care measures during such attacks.[7] To identify effective ways to enhance the self-care of this disease cohort, we also examined the factors that most significantly predicted their self-care and found that self-care knowledge and social support, although important, did not play the most significant role in predicting effective self-care. Rather, self-care confidence was the most influential factor (standardized path coefficient, $\beta= 0.386$, $p < 0.001$). [7] This finding implied that effective self-care interventions need to go beyond education and enable patients to be in control of their daily self-care. However, dialectic education, which only focuses on providing information, was most commonly used to enhance CHF self-care in our health care context, although an integrative review of interventions to promote self-care has indicated its suboptimal effects in changing self-care.[8]

Empowerment-based educational model has received more attention in diabetic self-care,[9] but its application in HF has been rather minimal. The philosophical underpinning of this approach is that patients are the primary decision makers in managing their own diseases.[10] Promoting effective self-care therefore, emphasizes the development of their confidence and inherent capacity to be in control of the required behavioral changes. [10] Central to this educational model is the use of a patient-centred collaborative approach to optimize the patients' participation and engagement in self-care decision making.[11] Education remains an essential component in this model, but emphasis is placed on using interactive teaching strategies and experiential learning to enable patients to translate complex health information to their own self-care practices. [11] More importantly, this model incorporates a goal-setting process to ensure that patients act autonomously and are motivated to manage their own disease. [12] This process begins by helping patients to identify their own self-care deficits and the associated health consequences. The patients are then, assisted in setting up their self-directed goal, and their beliefs, feelings and perceived challenges in goal attainment are ascertained. A collaborative effort is needed to maximize the number of choices available to the patients and to develop an action plan, with on-going support for problem resolution and goal adjustment. Previous studies have consistently identified the significant effects of empowerment-based education model in improving the self-confidence and self-efficacy of the patients in managing their chronic condition.[13-14]

Evidence from research on the effects of empowerment-based educational models on the self-care of HF patients has until recently been very limited. Shao et al[15] had a nurse deliver a single session of intensive education and assist patients in setting goals and formulating an action plan in a home environment. This was followed by four telephone follow-ups over 11 weeks to monitor goal attainment and provide help in problem resolution. The patients reported more positive changes in their self-care maintenance, self-care management and health-related quality of life (HRQL) after this 12-week intervention than those who received dialectic self-care education. Dickson et al[16] reported similar findings from a pilot study, that recruited lay health educators to deliver similar program activities (i.e., interactive teaching, goal setting and goal monitoring) in a group setting of 4 – 8 HF patients over four face-to-face biweekly sessions. This approach was found to be highly acceptable to the patients, who also appreciated the peer learning experience, even though it was a challenge to train lay health educators to deliver disease-specific empowerment-based education. Shively et al[17] involved nurses in delivering a similar group-based empowerment educational program over 15 weeks and also found positive effects on patients' HRQL. However, no self-care

outcomes were included in the evaluation. The PI had incorporated empowerment-based self-care education to a nurse-implemented transitional care for HF patients.[18] By delivering self-care education with this model in two weekly home visits and intensive telephone follow-ups, the HF patients had reported a significant improvement in self-care and HRQL. However, the multi-component nature of the transitional care precludes us from attributing such positive results to the sole effect of the empowerment-based education.

In conclusion, effective self-care is a cornerstone for the successful management of HF. Based on the findings of our previous HMRF project, the project team has identified a prompt need to improve the self-care of HF patients through modifying the two most significant predictors including self-care knowledge and self-care confidence. Empowerment-based educational model is recognized as an innovative approach to address these predictors, and there is increasing evidence to suggest its potential to improve the self-care knowledge, self-care maintenance and management and HRQL of HF patients. Indeed, this model which will be characterized by patient empowerment and tele-care, will be coherent with the strategies advocated by the health care service in Hong Kong for chronic disease management. In view of the inadequacy of current health care practices to address the self-care needs of the community-dwelling HF patients, there is an urgent need to examine the effects of the empowerment-based educational model in our health care setting.

Research Program

Purpose of study

The aim of this study was to evaluate the effects of a 12-week empowerment-based self-care education program that comprised five face-to-face sessions and telephone follow-up on self-care maintenance and management, self-care knowledge, self-care confidence, and HRQL in community-dwelling HF patients. Self-care domains were the primary outcomes, and the others, including self-care confidence, self-care knowledge, health-related quality of life, attendance to Accident and Emergence Department (AED) and hospital admission due to cardiac problem, are secondary outcomes. Cost-effectiveness analysis of the empowerment-based self-care program in improving self-care and HRQL and reducing cardiac-related A&E attendance and length of hospital stay was evaluated.

Hypothesis

The hypotheses for this study include:

1) Patients with HF who participated in the12-week empowerment-based self-care program will have better i) self-care maintenance; ii) symptom perception ii) self-care management; iii) self-care knowledge; iv) self-care confidence; v) HRQL, after the completion of the program and at 3 months thereafter than those who received didactic education.
2) Patients with CHF who participated in the 12-week empowerment-based self-care program will have i) fewer cardiac –related A & E attendance, ii) fewer cardiac-related hospital admission, iii) shorten total length of stay in hospital, and iv) more delayed time to hospital admission over the entire study period (.e., from baseline to 3 months upon the completion of the study interventions) than those who received didactic education.
3) The empowerment-based self-care program is more cost-effective than the didactic education to i) improve self-care maintenance; ii) improve symptom perception iii) improve self-care management; iv) improve quality-adjusted life year, v) reduce A&E attendance, and vi) reduce hospital stay over the entire study period (i.e., from baseline to 3 months upon the completion of the study interventions) than those who received didactic education.

Study design

This was a double-blind, randomized controlled trial, with HF patients randomly assigned to receive the empowerment-based self-care education program intervention or didactic education groups. To detect the immediate and longer-term effects of the empowerment-based self-care program, the outcome evaluation was measured at baseline, upon the completion of the study intervention, and at 3 months thereafter. Ethical approval was obtained from the Hospital Authority Clinical Research Ethics Committees.

Study Population

The study was conducted in the cardiac units of two regional hospitals in Hong Kong. Eligible patients were Chinese people aged 55 or over, who live in the community and have telephone access at home, with a confirmed medical diagnosis of HF of at least 6 months standing, and with New York Heart Association Class II-IV symptoms. The last two criteria exclude those who may not perform self-care because they lack illness experience or have too mild

symptoms. The exclusion criteria include having an impaired communication capacity or impaired cognitive function preventing participation in the program (as indicated by an Abbreviated Mental Test Score of ≤ 6). Power analysis, using the method of Hedeker et al[19] to estimate the sample size for a repeated measure study with attrition, was adopted. According to three similar studies, the effects size (Cohen's d) of self-care program based on the empowerment model for self-care maintenance, self-care management, self-care confidence, and HRQL at the 3-month study end-points were 1.24, 0.89, 0.45, and 0.54 respectively.[15-17] To be conservative, the current study aims to detect a small to medium effect size of 0.40 on the primary and secondary outcomes. Using the program RMASS, it is estimated that a sample size of 118 subjects per study arm would give the study 80% power at a 5% level of significance to detect an effect size of at least 0.4 on the primary and secondary outcomes between the two study arms at the post intervention time point (3 months), assuming there was no between-group difference on the outcomes at the baseline after randomization, a moderate auto-correlation of 0.4 within the intra-correlated outcomes and allowing for a 20% attrition rate.

Block randomization with a block size of 8, 10 or 12 was used to ensure an even distribution of subjects among the two study arms. The sequence of subject allocation will be generated in two stages. First, a computer will generate the sequence of block size to be used. For each chosen block size, the computer will then, randomly generate a sequence of subject allocation between the intervention and control groups. Subjects chronologically recruited to the study will be allocated to the study groups by the research nurse according to the computer generated sequence. SNOSE technique was used to keep the allocation concealment. The subjects was not informed of whether they participated in the test intervention or control intervention.

Study interventions

For the intervention group: the empowerment-based self-care education program

Subjects in the intervention group had participated in a 12-week empowerment-based self-care program, which was delivered by a cardiac nurse who has a baccalaureate degree in nursing and at least 3 years of clinical experience in cardiovascular nursing. The program will comprise five weekly 90-minute face-to-face sessions in small groups of 4-5 patients, followed by three weekly and two bi-weekly telephone follow-ups. Group teaching was used because the literature indicates patients appreciated the peer learning experience and its positive effect on improving motivation in behavioral changes.[16] To enhance the fidelity of the study intervention, a User Manual (**Additional Material 1**) which included the objectives and activity outlines for each face-to-face sessions, and the guidelines for conducting telephone follow-up care has been developed to guide the delivery of the intervention.

Face-to-face sessions

The five face-to-face sessions was delivered in the specialist clinics, and cover five major topics in CHF self-care, i) CHF manifestations and symptom monitoring, ii) dietary and fluid modification, iii) medication management, iv) recognition and management of deteriorated symptoms and v) activity and exercise. The educational content of each session complied with the international clinical guidelines and recommendations published by the British Heart Foundation and American Heart Association. The content has been validated by a multidisciplinary team that included cardiologists, an advanced practice nurse, nursing academicians in cardiovascular nursing and a dietician. The content has been used in a nurse-implemented transitional care conducted by the PI to educate CHF patients on how to manage the disease in a home setting.[18] The cardiac nurse received training from the project team, which included an advanced practice nurse, a nurse academician and a cardiologist, on international guidelines on CHF management and self-care, principles and skills in patient empowerment, and the methods used to deliver the study protocol.

Guided by empowerment-based philosophies in self-care education, each session will be incorporated with a five-step goal setting process to facilitate self-directed goal attainment.[12]

1) Each session began with discussions with the patients on self-care practices relevant to the particular topic area. The nurse used a number of counselling techniques, such as active listening, reflection, paraphrasing and empathetic understanding, to create a supportive and non-judgmental context in which the patients could disclose their self-care. (**Additional Material 1**)
2) The nurse then, delivered a structured educational presentation on the topic area. To optimize the effects of teaching, highly effective, colored pictures were used to illustrate the health information. Emphasis was placed on helping the patients to understand the link between the symptoms, the underlying mechanism of the disease, and the optimal self-care practice of CHF, so that they could more readily interpret their bodily cues of

symptom exacerbation and realize the importance of the recommended self-care.

3) The nurse helped the patients to identify their own self-care deficits by encouraging them to compare their usual self-care practice with the recommended health information, and highlighted the associated health effect of these behavioural discrepancies. After reflection, the nurse assisted the patients to set their self-care goals which was documented on a goal attainment form.
4) Skill building is followed during which interactive teaching strategies, such as role play or scenario-based group activities, will be used to enhance the patients' tactical and situational skills relevant to the topic of the session. The tactical skills focus on self-care skills in daily behaviours, while the situational skills focus on those required in challenging situations (e.g., dietary compliance when eating out or during festivals).[20]
5) The nurse encouraged the patients to discuss their feelings, concerns, perceived barriers and resources in achieving their goals. Each patient then set up individualized action plan for goal attainment. Resource notes relevant to the topic area in a concise writing style, with large-font print and visual depictions was provided to enable the patients to retrieve the required health information in a convenient and time-efficient manner. The nurse also helped to document the action plan with concise statements in the goal attainment form, which was kept by the patients as reminder.

Each session is began with a review of the key messages from and a discussion of the patients' progress in attaining the goals set in the previous session. Symptom monitoring, which enabled patients to detect early signs of fluid overload or inadequate cardiac output, was taught in the first session. This arrangement helped to increase the patients' understanding of the subsequently taught self-care practices, because fluid status and cardiac output serve as important indicators to guide the decision-making process for dietary modifications, diuretic adjustments and activity pacing. The patients will be taught to record their daily symptom status, including body weight, peripheral oedema and shortness of breath, on a self-monitoring form using simple methods (**Additional Material 2**), and emphasis was placed on teaching the patients to observe and interpret any changes. To help the patients to develop the habit of daily self-monitoring, the nurse reviewed their records for five consecutive weeks during the face-to-face sessions and provided them with feedback. As we found that the majority of CHF patients had difficulty in reading a needle-base bath scale in our previous HMRF project, a digital bath scale with an enlarged font display was provided to the intervention group. Because the main purpose of the study was to test the effects of an empowerment-based educational method, subjects in the control group also received the same weight scale. Indeed, the weight scale serves as a meaningful incentive to encourage subjects' participation in the study.

Telephone follow-up

The research nurse made three weekly and thereafter two bi-weekly telephone calls to the patients on the completion of the five face-to-face sessions. A telephone follow-up record has been developed to facilitate the process. The record documented the patient's clinical profile, CHF-related treatment (medication, diet and fluid prescription) and self-care goals and corresponding action plan identified in the face-to-face sessions. The nurse read this information before making the telephone call, so that she had clear focus on the monitoring and supporting self-care performance of each individual patient. During each telephone call, the nurse clarified any questions and concerns on self-care. Particular focus was placed on monitoring the level of goal attainment, identifying any challenges being encountered by the patients in implementing the action plan, and suggesting methods to resolve any self-care problems. Depending on the patients' progress, the goal and action plan was adjusted when necessary. Further health counselling that was relevant to individual patients' self-care needs were provided. After each telephone conversation, the nurse updated the telephone monitoring record by documenting the progress in goal attainment, any unresolved or new problems in self-care performance and the health counselling given. Such information helped to guide the discussions in subsequent telephone follow-ups.

For the control group: didactic education

Subjects in the control group received didactic education, which included a total of five 45-min health education sessions on the same topics as the intervention group. The nurse presented the health information to the patients, but no empowerment strategies, such as the five-step goal setting process, ongoing monitoring and patient-centered supportive interventions on goal attainment were offered. Upon completion of the education sessions, the nurse made three telephone calls over 7 weeks to each subject. The purpose of the telephone call will be to act as an

attention placebo. The nurse had general discussion on disease management with the patients and reminded them to follow the given health advice.

Study outcomes

The measures listed below were used to measure the outcome variables.

1) *<u>Self-Care Heart Failure Index (SCFHI; Chinese version)</u>*
   The 29-item SCHFI (Chinese version) [/21] was used to measure self-care maintenance, self-care management, symptom perception. A four-point response scale is used and each subscale score is transformed to 100 points, with a higher score representing a better self-care attribute. The SCHFI (Chinese version) is culturally relevant when used in elderly Chinese CHF patients in Hong Kong, with Cronbach's alpha of 0.93.

2) *<u>Dutch Heart Failure Knowledge Scale (DHFKS; Chinese version)</u>*
   The 15-item DHFKS (Chinese version)[/22] was used to measure self-care knowledge. These multiple-choice items determine the patients' knowledge of the disease, CHF symptom management and treatment. Good construct validity has been reported. Cronbach's alpha of the Chinese version is 0.72 and the content validity index is 0.94.[21]

3) <u>The Self-care Self-Efficacy Scale</u>
   The 10-item SCSES was used to measure the self-care efficacy of HF patients in performing self-care.[23] Each item was rated on a '1-5' Likert Scale and the total score is rescaled to '0-100' range, with higher score indicating higher level of self-confidence.

4) <u>Minnesota Living with Heart Failure Questionnaire (MLHFQ, Chinese version)</u>
   The 21-item MLHFQ (Chinese version)[24] will be used to measure the disease-specific HRQL of HF patients. The total score can range of 0 to 105, with higher scores indicating poorer HRQL. Cronbach's alpha is reported to be 0.95 and the construct, concurrent and convergent validity of the MLHFQ are supported by its significant correlation with the another reference measure of generic HRQL and depression among the Chinese CHF population.[24]

5) Accident and Emergence Department (AED) Attendance
   The AED attendance of the subjects after they have been randomized to the study group will be monitored up to a period of 6 months (i.e., from baseline to 3 months upon the completion of the study interventions). The information was traced from the clinical management system of Hospital Authority.

6) Hospital admission
   Hospital admission of the subjects after they have been randomized to the study group was monitored up to a period of 6 months (i.e., from baseline to 3 months upon the completion of the study interventions). The information was traced from the clinical management system of Hospital Authority. Information about the date of admission, the total length of hospital stay and the index diagnosis of admission was retrieved. The research nurse will also ask the subjects for any admission to the private hospital and collect the same information by patients self-report.

Statistical analysis

The data was double-entered for validation and analyzed on an intention-to-treat basis. Skewed variables would be appropriately transformed before being subjected to analyses. Baseline characteristics between the two arms of participants was compared using t-test, chi-square or Fisher's exact tests, as appropriate. For the self-care, self-care self-efficacy, heart failure knowledge and HRQL outcomes, generalized estimating equations (GEE) model will be used to compare the differential changes in each of the outcomes (self-care maintenance, self-care management, self-care confidence, self-care knowledge and HRQL) across the time-points T0 (baseline), T1 (post-test) and T2 (3 months after post-test) between the two study arms, with adjustment for potential confounding variables in order to obtain a more precise estimation of intervention effect. The potential confounding variables would selected on the basis of statistical incomparability at the baseline with p values < 0.25 for between-group differences[25]. GEE model can account for intra-correlated repeated measures data and accommodate missing data caused by incomplete visits

or drop-out, provided the data are missing at random[26] and thus is particularly suitable for intention-to-treat (ITT) analysis without the need to impute missing data. If the data is not missing at random, multiple imputation method would be used to impute missing data, using all available baseline characteristics as covariate. Predictive mean matching approach[27] which relies less on the parametric assumptions of the imputation models would be used. The episodes of accidental emergency department (AED) attendance and hospitalization was compared between the two groups using generalized linear model with the use of appropriate link function. The choice of the link function will be determined on the basis of the distributions of the above hospital utilization outcomes. Furthermore, Cox regression analysis will be performed to compare the time to hospital admission between the two.

Cost effectiveness analysis was performed on total cost incurred in both the empowerment-based self-care program (intervention) and dialectic education (control) as well as incremental cost-effectiveness ratios expressed as incremental cost per (1) an AED attendance avoided, (2) a day of hospitalization reduced, (3) a minimal clinically important difference (MCID) in SCFHI scores (self-care management, symptom perception, and self-care maintenance which showed significant between-group difference), and (4) a quality-adjusted life year (QALY) gained derived from MLHQ over the period from T0 to T2 (i.e., approximately 6-month time horizon) of the study. The component items of the total cost, including intervention, direct medical and societal costs are listed in Table 1. All the cost data involved will be expressed in Hong Kong dollars and valued on the starting date of the study from a health-care system perspective (i.e., all the medical costs will be valued on the basis of non-subsidized cost). All intervention, direct medical and societal costs incurred will be estimated per each participants using the method of Thompson & Barber[28] and the average cost difference between the intervention and control groups (intervention – control) was used to derive the incremental total cost (or total cost saved). Biased-corrected and accelerated bootstrapping method with 10000 replications [29] will be used to estimate a 95% confidence interval (CI) for the incremental total cost (or total cost saved). Mean differences in (1) episode of AED attendance and (2) total length of hospitalization during the period from T0 to T2 (approximately 6 months as well as (3) one MCID SCFHI at T2 (6 months) relative to T0 (the baseline) and (4) QALY between the two groups were adopted as the incremental effect measures. The above bootstrapping method was used to estimate 95% confidence intervals for the incremental effects. The bootstrapped 10000 pairs of incremental cost and each of the above effect measures data was plotted on a cost-effectiveness plane to graphically illustrate their uncertainties surrounding the cost-effectiveness ratios. Cost-effectiveness acceptance curves [30] were generated separately for each of the four types of cost-effectiveness evaluation outcomes to demonstrate the probability of cost-effectiveness of the empowerment-based self-care program over dialectic education at different thresholds for willingness-to-pay for saving an episode of AED attendance and a day of hospitalization as well as for MCID in SCFHI scores and a QALY gained

Ethics approval

Approval has been obtained from the Hospital Authority Clinical Research Ethics Committees. The study aim and procedure was explained to the patients in verbal and through written forms. Issues of voluntary participation, confidentiality and anonymity, and right to withdraw was emphasized. In particular, the subjects' was informed that they could refuse to participate or withdraw consent at any time without affecting the service they are entitled to receive. The subjects' name will not appear on any data record sheets and will be locked up in a secure location. Confidentiality of the subjects' personal data will be protected according to the Personal Data (Privacy) Ordinance. They can enjoy this right for the protection of their personal data, such as collection, retention, management, use (including analysis or comparison), non-disclosure, and erasure and/or in any way dealing with or disposing of any of their personal data in or for this study. Written informed consent will be obtained.

Impact and future direction

The detrimental consequences of HF not only hamper the quality of life of patients, but also strain already over-stretched hospital resources. As ineffective self-care behaviour has been consistently identified as a significant modifiable predictor of hospital admissions, enhancing patients' ability to manage the disease and treatment regimen is regarded as the cornerstone of a successful HF management model. However, the degree to which HF-related self-care enhancement interventions can benefit clinical outcomes is uncertain. The empowerment-based self-care programme proposed in this study is targeted at addressing this research gap. The unique program features to empower the patients to engage in the complex decision making of symptom recognition, interpretation and

response as well as self-care maintenance through goal-setting, patient-professional partnership and explicit methods and tools are central to modify their clinical outcomes. If the empowerment-based self-care programme is effective in enhancing the self-care ability and HRQL of HF patients in a cost-effective pattern, the intervention, together with the established materials (e.g. protocol, resource materials, and telephone follow-up records), can readily applied to clinical venues locally and beyond through knowledge translation process. The model of self-care enhancement can also be applied to primary care settings to empower CHF patients to manage the disease in the community. It can also be integrated to rehabilitation or transitional care service to enhance the patients' outcomes. Whereas international practice guideline advocates on self-care enhancement for this clinical cohort, this study can provide important insights to advance the corresponding evidence-based practice.

Table 1: Parameters of intervention cost, direct medical cost and social cost for cost-effective analysis

Parameters	Value (USD)	Reference
Intervention cost		
Empowerment-based self-care program		
• Staff training (20 hours)	$641	
• Staff cost (per patient; nursing time* hourly salary)[a]	6.83hrs x $21.8	Hospital Authority Pay Scale 2017 (point 17)
• Teaching Materials & weight scale (per patient)	$19.2	
Didactic education		
• Staff training (10 hours)	$320.5	
• Staff cost (per patient; nursing time* hourly salary)[b]	1.65 hr x $21.8	Hospital Authority Pay Scale 2017<br>(point 17)
• Teaching materials (per patient)	$14.1	Weight scale + teaching materials
Direct medical cost		
• AED attendance (per visit)	$126.9/ attendance	Fee and charges 2017, Hong Kong Hospital Authority
• Hospitalization without ICU care (per day)<br>• [all expense inclusive]	$600/day	
• Hospitalization with ICU care (per day)<br>• [all expense inclusive]	$2,948.7/ day	
• Post-discharge outpatient cost	$160.3 /visit	
• Specialist outpatient clinic	$152.6 visit	
• Private medical service relating to cardiac problems		Private hospital receipt
Societal Cost[c]		
• Productivity loss of caregiver who accompany patients to attend the intervention/ care of the patients	---	
• Productivity loss of the patients (e.g. absence from job duty) to attend the intervention/ due to the illness	---	
• Out-of-pocket cost from patient/ companion in attending the intervention (e.g. traveling expense)/ managing the disease.	---	

[a] nursing time / patient = {[90min/ session + 120min (preparation time)/ session]* 5 sessions/ 5 patients per group} + 40mintues/ telephone follow-up (including preparation and documentation time)* 5 times =360minute (6.83hours)

[b] nursing time/ patient = {[45min/ session + 30min (preparation time)/ session]*5 sessions/ 5 patients per group} + 8 minutes/ telephone follow-up * 3 times = 84minute (1.65 hour)

[c] values to be confirmed with the relevant parties (i.e. patient, caregiver and hospital administrative staff for venue rental)

References

1. Murray SA, Kendall M, Boyd K, Sheikh. Illness trajectories and palliative care. BMJ 2005; 330(7498):1007-1011.
2. Hospital Authority. Hospital Authority Statistical Report 2011/ 2012. Hong Kong: Hong Kong Hospital Authority; 2013.
3. Ma HM, Lum CM. Readmission of patients with congestive heart failure: the need for focused care. Asian J Gerontol Geriatr 2006; 1:59-60.
4. Van der Wal MJ, van Veldhuisen DJ, Veeger NJ, Rutten FH, Jaarsma T. Compliance with non-pharmacological recommendations and outcome in heart failure patients. Eur Heart J 2010; 31:1486-1493.
5. Riegel B, Lee CS, Dickson VV, Carlson B. An update on the Self-Care of Heart Failure Index. J Cardiovasc Nurs 2009; 24:485-497.
6. MaDonagh TA, Blue L, Clark AL, Dahlstrom U, Ekman I, Lainsack M et al. European Society of Cardiology Heart Failure Association Standards for delivering heart failure care. Eur J Heart Fail 2011; 13:235-241.
7. Yu DSF, Choi KC, Lee DFT, Yue S, Tsang KK. The mediating role of self-care confidence in the relationship between psychological attributes and self-care behaviors among heart failure patients. Eur J Heart Fail 2014; 16(S2):179-180.
8. Barnason S, Zimmerman L, Young L. An integrative review of interventions promoting self-care of patients with heart failure. J Clin Nurs 2011; 21:448-475.
9. Funnell MM, Tang TS, Anderson RM. From DSME to DSMS: Developing empowerment-based diabetes self-management support. Diabetes Spectr 2007; 20:221-226.
10. Feste C, Anderson RM. Empowerment: from philosophy to practice. Patient Educ Couns 1995; 26:139-144.
11. Anderson RM, Funnell MM. Patient empowerment: Myths and misconceptions. Patient Educ Couns 2010; 79:277-282.
12. Funnell MM, Anderson RM. Empowerment and self-management of diabetes. Clin Diabetes 2004; 22:123 - 127.
13. Anderson RM, Funnell MM, Aikens JE, Krein SL, Fitzgerald JT, Nwankwo R, Tannas CL, Tang TS. Evaluating the efficacy of an empowerment-based self-management consultant intervention: results of a two-year randomized controlled trial. Therapeutic Patient Education 2009; 1: 3-11.
14. Heinrich E, Schaper NC, de Vries NK. Self-management interventions for type 2 diabetes: a systematic review. European Diabetes Nursing, 2010; 7: 71-83.
15. Shao JH, Chang AM, Edwards H, Shui TL, Chen SH. A randomized controlled trial of self-management programme improves health-related outcomes of older people with heart failure. J Adv Nurs 2013; 69:2458-2469.
16. Dickson VV, Melkus GD, Katz S, Wong AL, Dillworth J. Cleland CM, et al. Building skill in heart failure self-care among community dwelling older adutls: Results of a pilot study. Patient Educ Couns 2014, 96:188-196.
17. Shively M, Kodiath M, Smith TL, Kelly A, Bone P, Fetterly L, et al. Effect of behavioral management on quality of life in mild heart failure: a randomized controlled trial. Patient Educ Couns 2005; 58:27-34.
18. Yu DSF, Lee DTF, Stewart S, Thompson DR, Choi KC, Yu CM. Impact of nurse-implemented transitional care for Chinese patients with chronic heart failure in Hong Kong: a randomized controlled trial. J Am Geriatr Soc. Forthcoming.
19. Hedeker D, Gibbons RD, Waternaux C. Sample size estimation for longitudinal designs with attrition: comparing time-related contrasts between two groups. J Educ Behav Stat 1999; 24:70-93.
20. Dickson VV, Riegel B. Are we teaching what patients need to know? Building skills in heart failure self-care. Heart Lung 2009; 38:253-261.
21. Yu DSF, Lee D, Thompson D, Woo J, Leung E. Assessing self-care behaviour of heart failure patients: Cross-cultural adaptation of two heart failure self-care instruments. Hong Kong Medical Journal 2010; 16 (Suppl 3), 13-16.
22. Chan WS. Self-care and self-care knowledge among Hong Kong older people with heart failure. [Dissertation]. Hong Kong: Chinese University of Hong Kong;
23. Riegel B, Lee CS, Dickson VV & Carlson B. An update on the self-care of heart failure index. Journal of Cardiovascualr Nursing, 24, 485-497.
24. Ho CC, Clochesy JM, Madigan E, Liu CC. Psychometric evaluation of the Chinese version of the Minnesota

Living with Heart Failure Questionnaire. Nurs Res 2007; 56:441-8.
25. Mickey, RM and Greenland, S. The impact of confounder selection criteria on effect estimation. Am J Epidemiol 1989; 129(1):125–137.
26. Singer JD, Willett JB. Applied longitudinal data analysis: modelling change and event occurrence. New York: Oxford University Press; 2003.
27. Little RJA: Missing-data adjustments in large surveys. Journal of Business & Economic Statistics 1988; 6: 287-96.
28. Thompson SG, Barber JA. How should cost data in pragmatic randomised trials be analysed? BMJ 2000; 320: 1197-200.
29. Efron B. Better bootstrap confidence intervals. Journal of the American Statistical Association 1987; 82: 171-85.
30. Fenwick E, O'Brien BJ, Briggs A. Cost-effectiveness acceptability curves: facts, fallacies and frequently asked questions. Health Economics 2004; 13: 405-15.

Additional Material 1

User Manual for the Empowerment-Based Self-Care Programme

<u>INTRODUCTION</u>

This User Manual is to guide the intervener to conduct the empowerment-base self-care program for patients with chronic heart failure. The purpose of the program is to develop patients' inherent capability and confidence to be in control of self-care practices required to management their cardiac disease. This 12-week program consists of five 90-minute face-to-face sessions over five weeks and thereafter, intensive telephone follow-up over the other seven weeks. The following figure outlines a brief graphical presentation of the empowerment-based self-care program.

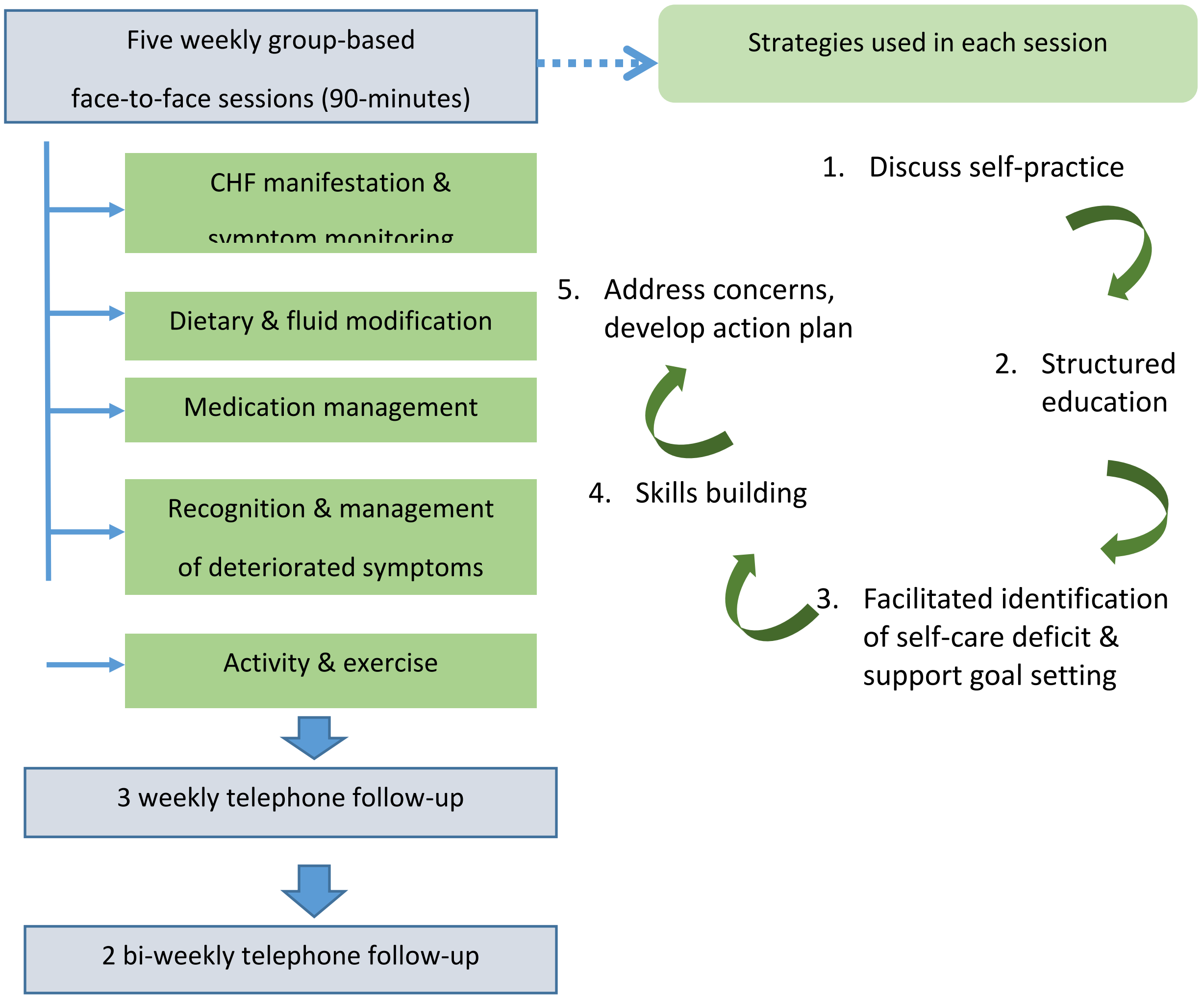

The manual consists of **three main sections**. The first section outline the counseling techniques used to deliver the program. The purpose of using such counseling techniques is to create a non-judgmental, accepting, empathetic and respectful atmosphere to encourage the patients to disclose their own self-care deficits and to work collaboratively with the health care professionals to set up realist self-care goal and action plan. The second section outlines the objectives, the equipment and the activities of the five face-to-face sessions. The last section outlines the activities of telephone follow-up.

SECTION 1: Counseling techniques for delivering the empowerment-based self-care program

In delivery the program, the intervener has to use the following counselling techniques appropriately in order to create a non-judgmental, accepting, empathetic and respectful atmosphere to encourage the patients to disclose their own self-care deficits. The intervener has to maintain an 'equal' relationship with the patients and to be empathetic to the patients' concern and resource. This approach would encourage patients to work collaboratively with the intervener in setting up realist self-care goal and action plan.

1) Being supportive in assisting patients to identify inadequacy in self-care and being genuinely accepting and non-judgemental to the inadequate self-care practice.
2) Being patient-centred in identifying the challenges faced by patients in performing self-care. Using active listening to identify concerns, issues and beliefs that may hinder effective self-care. [e.g. "Tell me what is hardest for you to follow the dietary modification?'].
3) Being a collaborator rather than a director in assisting patients to work through ambivalence. Providing health advice and encouraging them to make choices as to what should change and how change should take place. [e.g. "What support do you think will help you most to comply with the self-care recommendation?"].
4) Use reflection and paraphrasing to validate patients' feelings and their capacity to deal with the problem [e.g. "What would you like to try?"].
5) Showing respect and genuine belief in the patients' ability to mobilize resource and make the changes.
6) No direct confrontation techniques will be used.

SECTION 2: Outlines of the five face-to-face sessions

List of the five face-to-face sessions

Session number	Topic area
Session One	CHF manifestation and symptom monitoring
Session Two	Dietary and fluid modification
Session Three	Medication management
Session Four	Recognition and management of deteriorated symptoms
Session Five	Activity and exercise

Session One: CHF manifestation and symptom monitoring

Objectives

1. Patients have clear information about the purpose, schedule and activities of the self-care program.
2. Patients understand heart failure and how the symptoms are related to the disease manifestation.
3. Patients are able to monitor for the common heart failure symptoms which indicate fluid overload or unmet metabolic needs.

Equipment:

1. PowerPoint projector
2. PowerPoint Slides for structured education on heart failure, its symptom manifestation and symptom monitoring.
3. Resource notes on heart failure and symptom monitoring.
4. Equipment for skill buildings
   - Paper cards with colored picture to show various symptoms.
   - Bath weight scale with large-font digital display.
   - Measuring tape with large-font scale
   - Symptom self-monitoring charts and pencils.

Activities:

1. Ice-breaking & introduce the run-down of the overall empowerment-based self-care program.
2. Solicit patients' views about heart failure and the associated symptom manifestations.
   - Patients take turn to describe what heart failure is. Create a non-judgmental atmosphere and encourage them to present their views.
   - Game 1:
     - Each patient will be provided with a A4-size white card.
     - Place card boards with colored picture of various symptoms including (fatigue, shortness of breath, weight gain, edema of the ankle, feet or abdomen, headache, nausea, muscle cramps, dizziness, loss of appetite, blurring of vision, chest tightness, chest pain, etc).
     - Patients will have two minutes to pick the cards with HF symptoms and stick on put on their A4 card.
     - Patients will be asked to explain why they make the selection.
3. Structured education on the following topics:
   - What is heart failure.
     - Uses 'The animation of heart failure" by the American Heart Association (http://watchlearnlive.heart.org/CVML_Player.php?moduleSelect=hrtflr).
     - Highlight failing of the myocardium pump to maintain adequate metabolic needs and normal venous blood flow.
   - Symptom manifestations of heart failure.
     - Highlights that the symptoms occur as a result of venous congestion and inadequate myocardial blood supply, and thereby explain the importance of symptom recognition.
   - PowerPoint-guided story telling (The story depicts scenes of a HF patients (Sum Kor) accurately integrating symptom monitoring on edema, body weight, fatigue and shortness of breath to everyday life. The story will illustrate the common barriers to implement this self-care and the corresponding coping methods.
4. Identification of self-care deficits:
   - Facilitate the patients to compare
5. Skill building
   - Symptom monitoring (body weight, ankle edema, fatigue and shortness of breath) and easy charting on the symptom monitoring form
   - Skills on observing the changes in symptoms.
6. Facilitate patients to take turn to mention how they incorporate symptom monitoring to daily routine.
   - Encourage patients to brainstorm how to achieve their identified goal. Provide as many options as possible.
   - Encourage patients to discuss their concerns and anticipated barriers.
   - Encourage patients to identify resource (person/ equipment) which could help.
   - Suggest resolving method and facilitate peer sharing.
   - Summarize the actions for goal attainment for each patient, and assist patients to document on the goal attainment booklet.

7. Distribute the resource note and the bath weight scale.

Session Two: Dietary and fluid modification

Objectives:

1. Patients understand the role of dietary and fluid modification on preventing cardiac overload.
2. Patients are able to select the food and cooking methods which are appropriate for heart failure in regular days or during special events (e.g. Chinese New Year Festival, attending a banquet) .
3. Patients are able to adjust the dietary and fluid intake in responding to the changing symptom condition.

Equipment:

1. PowerPoint projector
2. PowerPoint Slides for structured education on dietary and fluid modification for heart failure
3. Resource notes on dietary and fluid m monitoring.
4. Equipment for skill buildings
   - Paper cards with colored picture of various food and seasoning
   - Paper cards with colored picture of various cooking methods
   - Food labels
   - Restaurant menus
   - Fluid intake monitoring chart

Activities

1. Welcome patients and review the symptom monitoring charts. Discuss how well patients conduct the action plan for goal attainment in the previous week.
2. Facilitate patients to take turn to describe their current self-care practice on dietary and fluid modification. Focus the discussion on food selection, cooking methods, use of seasoning, fluid restrictions.
3. Structured education on the following topics using the PowerPoint Slides.
   - The influence of sodium and fluid retention on heart function. .
   - Appropriate methods to maintain dietary and fluid modifications for heart failure, with emphasis on selection of low sodium food and seasonings, correct cooking methods, fluid intake monitoring.
   - Methods to identify sodium content from food label
   - Ways to adjust dietary and fluid intake according to the symptom status.
   - PowerPoint-guided story telling (The story depicts scenes of a HF patients (Sum Kor) who accurately follow the dietary and fluid modifications in everyday life. The story illustrates the common barriers to implement this self-care and the corresponding coping methods.
4. Identification of self-care deficits:
   - Facilitate the patients to compare themselves with “Sum Kor” regarding the dietary and fluid modification.
     - Facilitate the patients to think about what will happen if they do not comply to the diet and fluid modification.
     - Recall the information in the structured education and highlight the health consequence of the associated self-care deficit.
   - Support the patients to set their self-care goals on dietary and fluid modification.
   - Document the identified goals on the goal attainment booklet.
5. Skill buildings
   - Game 1: By using the picture cards of various food, seasonings and cooking methods, each patient are required to preparing two dishes of low sodium diet. The peer will comment on whether the dishes are appropriate for heart failure. They will also vote for the most favorite dish.
   - Game 2: Patients will be divided into two groups, each group is provided with food labels of five different types of food. They were required to pick up two for which the sodium content is regarded as appropriate for heart failure patients.
   - Game 3: Patients will be divided into two groups. Each group is provided with a restaurant menu. Tell the patients that they are having dinner with four family members who prefer tasty food. The patients are required to practise assertively on how to participate in the dish selection and pick appropriate diet to prevent excessive sodium intake.

6. Facilitate patients to take turn to mention how they incorporate dietary and fluid modification to everyday life.
    - Encourage patients to brainstorm how to achieve their identified goal. Provide as many options as possible.
    - Encourage patients to discuss their concerns and anticipated barriers.
    - Encourage patients to identify resource (person/ equipment) which could help.
    - Suggest resolving method and facilitate peer sharing.
    - Summarize the actions for goal attainment for each patient, and document the action plan on the goal attainment form.
7. Distribute the resource note on dietary and fluid modification, in which patients can find a pictorial guide on low sodium food and high sodium food, a pictorial guide of low-sodium diet from menus of Chinese restaurant. Assist the patients to document the action plan on the goal attainment form.

Session 3: Medication management

Objectives:

1. Patients are able to identify the indications, common side effects and safety considerations of the prescribed heart failure medications.
2. Patients are able to identify the principle and method of flexible diuretic regime as prescribed in the involved study settings.
3. Patients are able to identify medication safety tips for medication usage, storage and refill.

Pre-session preparation

Need to check the current medication record of the patients in the group from the baseline clinical data so as to tailor the education content.

Equipment:

1. PowerPoint projector
2. PowerPoint Slides for structured education on medication management for heart failure
3. Resource notes on medication management for heart failure.
4. Equipment for skill buildings
    - Paper cards with colored picture of various medication storage methods
    - Pill boxes

Activities

1. Welcome patients and review the symptom monitoring charts. Discuss how well patients conduct the action plan for goal attainment in the previous weeks.
2. Solicit patients' views about the role of medications in heart failure management and their current practice on medication management.
    - Patients take turns to describe the purpose of taking the current medications.
    - Patients take turns to describe the methods they used to prevent medication errors (e.g. skip dose or taking the wrong medications).
    - Patients take turns to describe how they adjust the diuretics according to their symptom status.
3. Structured education on the following topics using the PowerPoint Slides.
    - The roles of heart failure medications in disease management.
    - The indications, common side effects and safety considerations of the heart failure medications (focused on those which are currently prescribed for the participants in the group)
    - The principles and methods of taking flexible diuretic regimen.
    - Quick tips to enhance medication compliance.
    - Medication safety tips.
    - PowerPoint-guided story telling (The story depicts scenes of a HF patients (Sum Kor) who uses appropriate methods to adjust diuretic regimen. He also uses various effective methods to prevent pitfalls in committing medication errors.
4. Identification of self-care deficits:
    - Facilitate the patients to compare themselves with "Sum Kor" regarding medication management. .
        - Facilitate the patients to identify the discrepancies, if any, in the medication management.

- Recall the information in the structured education and highlight the potential pitfalls they may encountered if they continue their usual medication management practice.
- Support the patients to set their self-care goals on medication management.
- Document the identified goals on the goal attainment booklet.

5. Skill buildings
   - Scenario-based discussion: Presenting two scenarios which illustrate the different symptoms of fluid overload. Each patient has a dummy of diuretic. They have to make decision about how to adjust the dose.
   - Scenario-based discussion: Presenting three scenarios on medication storage and medication taking behaviors. Patients are required to explain whether the behaviors are correct or not.
   - Game 1: Each patient is provided with three HF medications (dummies of different color) as their usual medication. They are then provided with a new medication regimen after a clinic visit in which one or two medications have been changed. They are required to demonstrate how to update the medication storage with the new and old medications.
   - Game 2: Each patient is provided with a multi-compartment pill organizer. They are provided with the dummy medications which assimilates their current medication regimen. Each of them is required to demonstrate how to correctly fill the pill organizer.
6. Facilitate patients to take turn to mention how they incorporate the recommended medication management strategies in their daily life.
   - Encourage patients to brainstorm how to achieve their identified goal. Provide as many options as possible.
   - Encourage patients to discuss their concerns and anticipated barriers.
   - Encourage patients to identify resource (person/ equipment) which could help.
   - Suggest resolving method and facilitate peer sharing.
   - Summarize the actions for goal attainment for each patient, and document the action plan on the goal attainment form.
7. Distribute the resource note on medication management. Assist the patients to document the action plan on the goal attainment form.

Session 4: Recognition and management of deteriorated symptoms

Objectives

1. Patients are able to identify the early cues of deterioration of heart failure.
2. Patients are able to make correct decision about how to respond to the deteriorated symptoms.
3. Patients are able to identify methods to evaluate the effectiveness of their remedies for the deteriorated symptoms.

Equipment:

1. PowerPoint projector
2. PowerPoint Slides for structured education on recognition and management of deteriorated heart failure symptoms
3. Resource notes on recognition and management of deteriorated heart failure symptoms.
4. Equipment for skill buildings
   - Paper cards with colored picture to show bodily cues which may or may not indicate deteriorated heart failure.
   - Paper cards with colored picture to show various ways to manage CHF symptom exacerbation. Some methods are correct and some are irrelevant.

Activities

1. Welcome patients and review the symptom monitoring charts. Discuss how well patients conduct the action plan for goal attainment in the previous weeks.
2. Facilitate patients to describe the current practice in manage deteriorated heart failure symptoms.
   - Game:
     - Each patient will be provided with a A4-size white card.
     - Place card boards with colored picture to show bodily cues which indicate deteriorated heart failure on the table.
     - Patients will have two minutes to pick the cards which indicate deteriorated heart failure

symptoms on the A4-size card.

- Patients have to explain why they pick the cards and how they response to those bodily changes.

3. Structured education on the following topics using the PowerPoint Slides.
   - The bodily cues which indicate the deterioration of heart failure.
   - Methods to increase ones' awareness to deteriorated symptoms.
   - Early remedies to reverse the deteriorated symptoms through dietary, activity and diuretic adjustment.
   - Methods to monitor and evaluate the effectiveness of the remedies for deteriorated symptoms.
   - Ways to make decision for seeking medical attention for deteriorated heart failure symptoms.
   - Health consequence for delayed remedy or care seeking for the disease deterioration.
   - PowerPoint-guided story telling (The story depicts scenes of a HF patients (Sum Kor) who recognizes early deteriorated heart failure symptoms and demonstrate appropriate actions to cope with the symptoms. The scenario also illustrates how to cope with the common psychosocial barriers which hinder prompt self-care management.
4. Identification of self-care deficits:
   - Facilitate the patients to compare themselves with "Sum Kor" regarding recognition and responses to bodily cues which indicate the deterioration of heart failure.
     - Facilitate the patients to identify the discrepancies, if any, in how to monitor for and response to deteriorated heart failure symptoms.
     - Recall the information in the structured education and highlight the detrimental health consequence of their self-care deficit in managing the symptom deterioration.
   - Support the patients to set their self-care goals on medication management.
   - Document the identified goals on the goal attainment booklet.
5. Skill buildings
   - <u>Game:</u> Divide patients into two groups. Presenting four scenarios which illustrate different symptoms representing deteriorated heart failure. For each scenario, each group has to identify the paper cards which illustrate the correct ways to manage CHF symptom exacerbation. They have to describe how they would determine the actions as effective to overcome the symptoms. Provide feedback to patients accordingly.
6. Facilitate patients to take turn to mention how they incorporate the recommended medication management strategies in their daily life.
   - Encourage patients to brainstorm how to achieve their identified goal. Provide as many options as possible.
   - Encourage patients to discuss their concerns and anticipated barriers.
   - Encourage patients to identify resource (person/ equipment) which could help.
   - Suggest resolving method and facilitate peer sharing.
   - Summarize the actions for goal attainment for each patient, and document the action plan on the goal attainment form.
7. Distribute the resource note on recognition and management of deteriorated heart failure. Assist the patients to document the action plan on the goal attainment form.

<u>Session 5: Activity and Exercise</u>

<u>Objectives:</u>

1. Patients will be able to identify the advantages of maintain physically active on energy level and cardiac functions.
2. Patients will be able to describe how to maintain a safe physical activity level through self-monitoring methods.
3. Patients will be able to identify how to pace the activity in responding to the bodily cues and when to stop the exercise or activity for the sake of safety.
4. Patients will be able to identify type of activities and exercise which are fit for their cardiac condition and overall health status.

<u>Preparation</u>

Remind patients to wear sport shoes to attend this session the day before.

Equipment:

1. PowerPoint projector
2. PowerPoint Slides for structured education on how to maintain a physically active lifestyle in living with heart failure.
3. Videos on how to carry out brisk walking with warm up and cool down phases.
4. Resource notes on how to maintain a physically active lifestyle for heart failure patients.
5. Equipment for skill buildings
6. Paper cards with colored picture to indicate relevant or irrelevant bodily cues which indicate activity intolerance.

Activities

1. Welcome patients and review the symptom monitoring charts. Discuss how well patients conduct the action plan for goal attainment in the previous weeks.
2. Facilitate patients to take turn to talk about their current physical activity or exercise habit, and to discuss the precautions, if any, they have to take during physical exertion.
3. Structured education on the following topics using the PowerPoint Slides.
    - The advantages of maintaining a physically active lifestyle in patients with heart failure and the detrimental health impact of a sedentary lifestyle.
    - What are the appropriate activities and exercise for heart failure patients
    - The importance of warm up and cool down exercise for heart failure patients.
    - The principles of activity pacing in everyday life.
    - Simple methods to evaluate whether the activity intensity is appropriate (e.g. able to carry out conversation during activity).
    - The bodily cues which indicate a need to slow down or stop the activities or exercises.
    - PowerPoint-guided story telling (The story depicts scenes of a HF patients (Sum Kor) who have successfully maintained an active lifestyle exercise in daily life. The story illustrates how he performs activity pacing according to the bodily cues and how he determines the need to stop the activity for rest.
4. Identification of self-care deficits:
    - Facilitate the patients to compare themselves with “Sum Kor” regarding physical activity level and the precautions taken in performing activity or exercise.
        - Facilitate the patients to identify the discrepancies, if any, in how to incorporate a physically active lifestyle in living with heart failure.
        - Recall the information in the structured education and highlight the detrimental health consequence of their self-care deficit in managing physically active.
    - Support the patients to set their self-care goals on medication management.
    - Document the identified goals on the goal attainment booklet.
5. Skill buildings
    - Video display: Display a video on brisk walking with warm up and cool down phases.
    - Return demonstration on the brisk walking with warm up and cool down.
    - Brainstorming: Facilitate the patients to brainstorm the physical activities or exercise which are enjoyable and fit with their activity tolerance level. Provide them with as many as options as possible.
    - Game: Divide patients into two groups. Each of the group has to pick up the picture cards which indicate a bodily cues to indicate activity intolerance or conditions which are contradictory to do physical activity within one minute.. The group has more correct card will be the winners. Providing feedback to the participants accordingly.
6. Facilitate patients to take turn to mention how they incorporate the recommended medication management strategies in their daily life.
    - Encourage patients to brainstorm how to achieve their identified goal. Provide as many options as possible.
    - Encourage patients to discuss their concerns and anticipated barriers.
    - Encourage patients to identify resource (person/ equipment) which could help.
    - Suggest resolving method and facilitate peer sharing.
    - Summarize the actions for goal attainment for each patient, and document the action plan on the goal attainment form.
7. Distribute the resource note on activity and exercise in which a pictorial guide on how to perform warm up, brisk walking and cool down are included. Assist the patients to document the action plan on the goal attainment form.

SECTION 3: Activities during telephone follow-up

Upon the completion of the five face-to-face sessions, the intervener has to give three weekly and thereafter two bi-weekly telephone calls to the patients. The main purpose of the telephone follow-up is to monitor patients' progress on goal attainment and to support their implementation of the action plans in their home environment. Efforts have to be placed on identifying patients' concerns, questions, and challenges encountered in implementing the self-care, and to suggest resolving methods. Further health counseling has to be provided, with emphasis placed on helping patients to translate the knowledge and skills taught in the face-to-face session to their own real life situation. Depending on the patients' progress, adjust the self-care goal and action plan accordingly.

Followings are the activity guide for the intervener before, during and after making the telephone call.

i) Before making the telephone call

- Please read the information on the telephone monitoring record on which you can find the clinical profile, CHF related information (medication, diet and fluid prescription) as well as the self-care goals and corresponding action plan. Such information can provide you with a clear focus in supporting and monitoring self-care performance of each individual patient.

ii) During the telephone follow-up

- Ask the patients how well they are implementing the action plan for goal attainment. Identify any improvement in behavioral changes. Provide encouragement and praise.
- Review the level of goal attainment. Communicate explicitly with the patients about the self-care goal which they have achieved. Encourage and facilitate patients to identify the benefits associated with the positive behavioral changes in self-care.
- Identify their concerns, problems and difficulties encountered by the patient in implementing the action plan. Discuss with the patients the possible resource can help to overcome the difficulties.
- Collaborate with the patients to identify any resolving methods to overcome the challenges. Recall the knowledge and skills taught in the face-to-face session, and provide suggestion on how such knowledge and skills can be applied to their real life situation.
- Remind the patients to read the relevant resource notes provided in each face-to-face session.
- Adjust the self-care goal if necessary and refine the action plan.

iii) Immediate after the telephone follow-up

- Update the telephone monitoring record and document the following information:
  - Progress of goal attainment
  - Any unresolved and new problems in self-care
  - The health counseling being provided

Additional Material 2

Self-Monitoring Form

1. **Rate your breath:**

On your left hand side, there is a '0-5' rating scale to mark your **level of shortness of breath during normal daily activity**, with '1' represents "no shortness of breath at all " and '5' represents "Shortness of breath as bad as can be". Please put a stick to represent your level of shortness of breath for each calendar day.

	Calendar Day						
	**1**	**2**	**3**	**4**	**5**	**6**	**7**
Shortness of breath as bad as can be → 5							
4							
3							
2							
1							
No shortness of breath → 0							

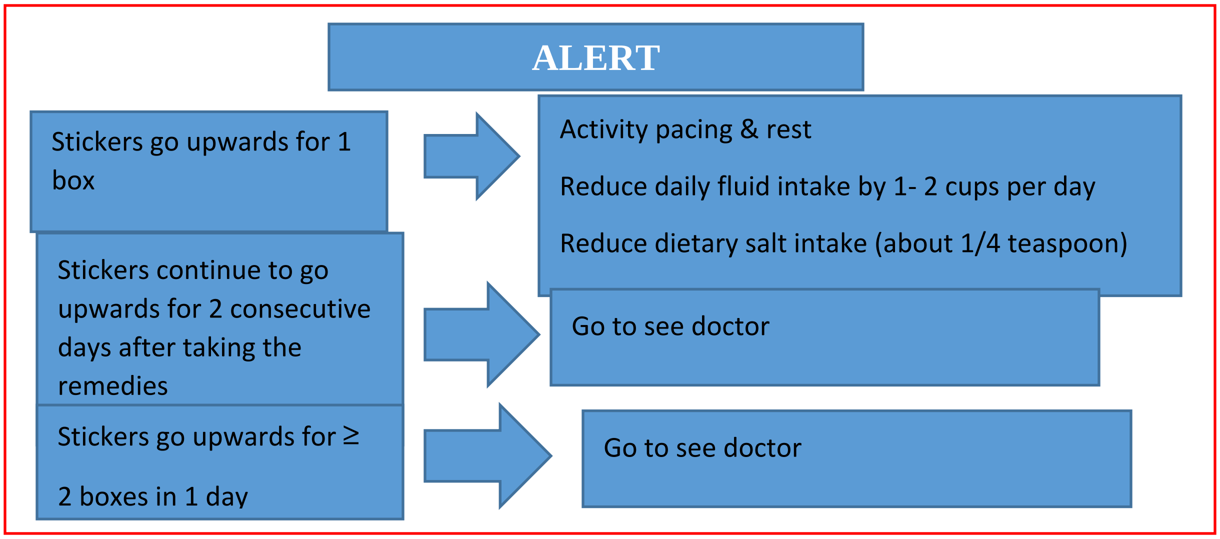

2. **Rate your swelling**

On your left hand side, there is a '0-5' rating scale to mark your **level of swelling over the feet, hands or belly** , with '1' represents "no swelling at all " and '5' represents "swelling as bad as can be". Please put a stick to represent your level of swelling for each calendar day.

Swelling as bad as can be → 5

No swelling → 0

	Calendar Day						
	**1**	**2**	**3**	**4**	**5**	**6**	**7**
5							
4							
3							
2							
1							
0							

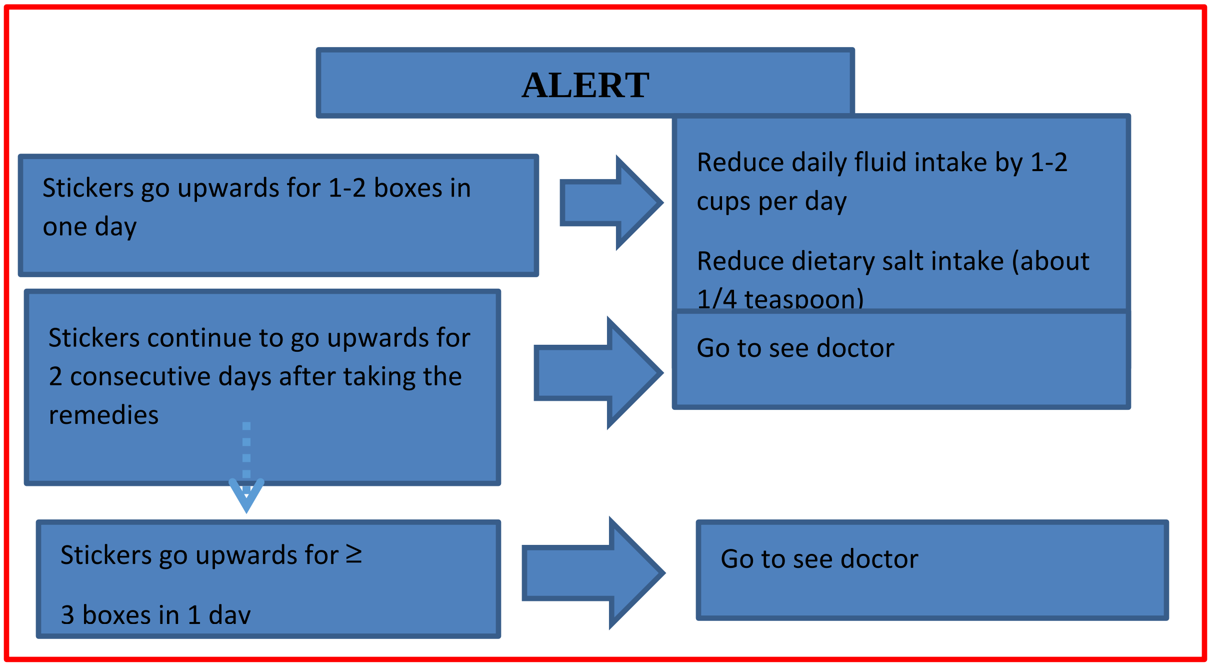

3. **<u>Rate your body weight</u>**

On your left hand side, there is a '0-5' rating scale to mark your **level of swelling over the feet, hands or belly** , with '1' represents "no swelling at all " and '5' represents "swelling as bad as can be". Please put a stick to represent your level of swelling for each calendar day.

		Calendar Day						
Nurse sets the scale according to the patient's baseline body weight (scale: a pound/box)		**1**	**2**	**3**	**4**	**5**	**6**	**7**
	136lb							
	135lb							
	134lb							
	133lb							
	132lb							
	131lb							
	130lb							
	129lb							
	128lb							
	127lb							
	126lb							
	Baseline(e.g. 125lb)							

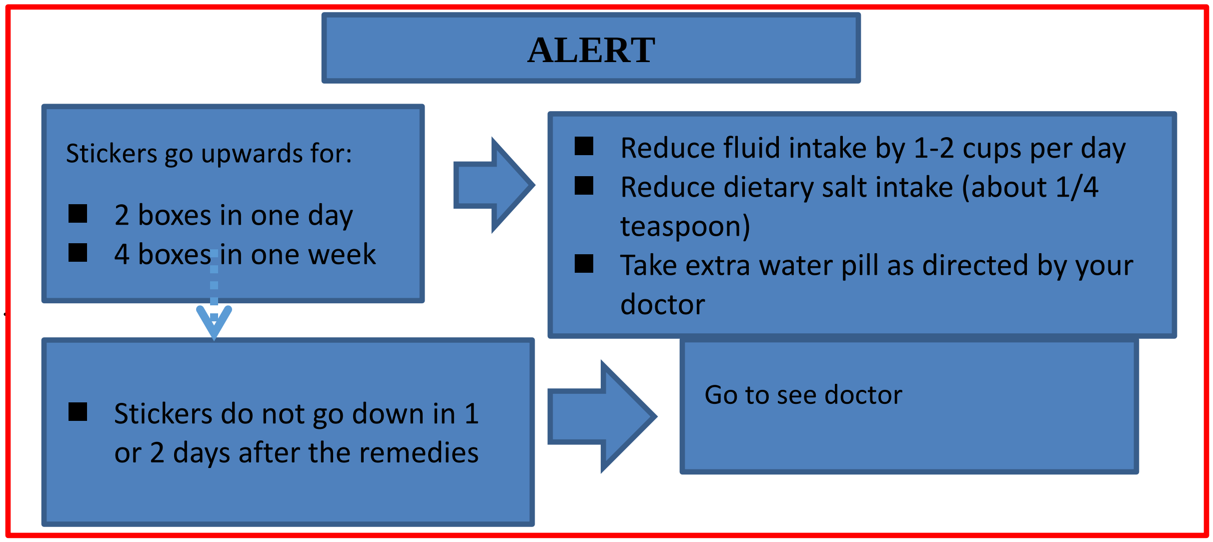

Supplement: Supplement 1. — Trial Protocol [file jamanetwopen-e225982-s001.pdf]
