# Supplementary material for: Effectiveness and Cost-effectiveness of an Empowerment-Based Self-care Education Program on Health Outcomes Among Patients With Heart Failure: A Randomized Clinical Trial
Source: JAMA Netw Open. 2022 Apr 5;5(4):e225982. doi: 10.1001/jamanetworkopen.2022.5982 (PMC8984788; doi:10.1001/jamanetworkopen.2022.5982)
Supplement: Supplement 2. — eMethods. Description of Economic Evaluation eTable 1. Parameters of Intervention Cost, Direct Medical and Social Cost for Cost-effectiveness Analysis eTable 2. Parameters Definition for Cost-effectiveness Model eFigure 1. Acceptance Curve of Empowerment and Education Control – Quality-Adjusted Life-year eFigure 2. Acceptance Curve of Empowerment and Education Control – Symptom Perception eFigure 3. Acceptance Curve of Empowerment and Education Control – SCHFI Management [file jamanetwopen-e225982-s002.pdf]

Supplementary Online Content

Yu DS-f, Li PW-c, Li SX, Smith RD, Yue SC-S, Yan BPY. Effectiveness and cost-effectiveness of an empowerment-based self-care education program on health outcomes among patients with heart failure: a randomized clinical trial. *JAMA Netw Open*. 2022;5(4):e225982. doi:10.1001/jamanetworkopen.2022.5982

eMethods. Description of Economic Evaluation

eTable 1. Parameters of Intervention Cost, Direct Medical and Social Cost for Cost-effectiveness Analysis

eTable 2. Parameters Definition for Cost-effectiveness Model

eFigure 1. Acceptance Curve of Empowerment and Education Control – Quality-Adjusted Life-year

eFigure 2. Acceptance Curve of Empowerment and Education Control – Symptom Perception

eFigure 3. Acceptance Curve of Empowerment and Education Control – SCHFI Management

This supplementary material has been provided by the authors to give readers additional information about their work.

© 2022 Yu DS-f et al. *JAMA Network Open.*

eMethods. Description of Economic Evaluation

Utility

We conducted four different economic evaluation models for different indicators of utility; one for QALYS and two sub scales of the SCHFI (symptom perception and management) as measurements of utility within the economic model. For the two sub scales of the SCHFI were taken as a unit of MCIDs gained between baseline and 6-month follow-up in both groups. QALYS were estimated using the MLHF using an algorithm to estimate EQ5D-5L utility score. None of our sample died during the follow-up period of the economic evaluation, so no adjustments to QALYS were made to account of death.

Costs

The component items of the total cost, including intervention, direct medical and societal costs are listed in the eTable 1. All the cost data were estimated in Hong Kong Dollars (HKD) and converted into the 2021 U.S. Dollars (1USD = 7.8 HKD) for reporting and valued on the starting date of the study from a health-care system perspective. The intervention cost comprised of three components including i) the staff training cost (with reference to the market price for professional training to nurses), ii) the cost of care in terms of the time spent with each patients (in hours) in delivering the interventions multiplied by the nurses' salary according to the payscale of the Hospital Authority, iii) the cost of training materials and weight scale for each patients, and iv) the venue rental for delivering the intervention.

The direct medical cost covered the hospital service utilization including emergence room utilization, outpatient cost, inpatient cost with and without the care from intensive care units, and post-discharge rehabilitation care. All the medical costs were valued on the basis of non-subsidized index cost in the health care system operated by the Hong Kong Hospital Authority which is a statutory body providing public hospital and related rehabilitation service to patients through hospitals, day hospitals, specialist clinic and general outpatient clinic. It covers 90 % of in-patient care and 32% of outpatient treatment within Hong Kong. The index cost is package charge which covers medications and diagnostic procedures.

The social cost covered the participants' or their caregivers' days of productivity lost or out-of-pocket expense during the study period in managing the disease and attending the intervention.

Health status

Our economic evaluation contained three different health status that participants could enter at the end of the study period; non-event, hospital admission relating to cardiac problem, emergence room attendance relating to cardiac problem. These health status were assessed at 6-month follow-up based on the data from the Hospital Authority electronic health record if patients were admitted to the public healthcare sector. For those who admitted to private healthcare sector, self-report method was used for assessment. Utility, costs and probability for each corresponding health status were estimated based on our study's data. We modelled utility, cost and transitional probability as distributions; utility was distributed as a beta-distributions for QALYS and normal distribution for subscales of SCHIFI, costs were estimated using gamma-distributions found to be heavily skewed, with many patients only have costs due to intervention/control costs. Health status transitional probability were estimated using fixed proportions (probability of being in each health status). Details of distributions for transitional probability, costs and utility can be found in eTable 2.

Incremental cost effectiveness ratio

The analytical time horizon was approximately 6 months (from baseline to 3 months post-test). We developed decision-tree analytical models with a Markov cohort simulation to incorporate the cost- and health-related outcomes for cost-effectiveness analysis. Using the education group as the reference group, several incremental cost-effectiveness ratios (ICERs) were computed to determine the potential cost-effectiveness of the empowerment-based self-care program.

The ICER is calculated as the ratio between the cost difference of both interventions and the difference of mean utility between the two interventions. A negative ICER indicating complete domination of the empowerment intervention (cost-saving and effective in increasing utility) compared to the control group. Cost effectiveness was also considered to be met for the empowerment intervention if the ICER was underneath a willingness-to-pay (WTP), in this cases used 1.0 GDP per capital of Hong Kong (359,164HKD, $46,081 USD). To account for the effects of uncertainties from the model we ran 10,000 bootstrapped Monte Carlo iterations and calculated confidence intervals of ICERs using bootstrap percentile methods.

eTable 1. Parameters of intervention cost, direct medical cost and social cost for cost-effectiveness analysis

Parameters	Value (USD)	Reference
Intervention cost		
Empowerment-based self-care program		
• Staff training (20 hours)	$641	
• Staff cost (per patient; nursing time* hourly salary)[a]	6.83hrs x $21.8	Hospital Authority Pay Scale 2017 (point 17)
• Teaching Materials & weight scale (per patient)	$19.2	
Didactic education		
• Staff training (10 hours)	$320.5	
• Staff cost (per patient; nursing time* hourly salary)[b]	1.65 hr x $21.8	Hospital Authority Pay Scale 2017 (point 17)
• Teaching materials (per patient)	$14.1	Weight scale + teaching materials
Direct medical cost		
• AED attendance (per visit)	$126.9/ attendance	Fee and charges 2017, Hong Kong Hospital Authority
• Hospitalization without ICU care (per day) • [all expense inclusive]	$600/day	
• Hospitalization with ICU care (per day) • [all expense inclusive]	$2,948.7/ day	
• Post-discharge outpatient cost	$160.3 /visit	
• Specialist outpatient clinic	$152.6 visit	
• Private medical service relating to cardiac problems		Private hospital receipt
Societal Cost[c]		
• Productivity loss of caregiver who accompany patients to attend the intervention/ care of the patients	---	
• Productivity loss of the patients (e.g. absence from job duty) to attend the intervention/ due to the illness	---	
• Out-of-pocket cost from patient/ companion in attending the intervention (e.g. traveling expense)/ managing the disease.	---	

[a] nursing time / patient = {[90min/ session + 120min (preparation time)/ session]* 5 sessions/ 5 patients per group} + 40mintues/ telephone follow-up (including preparation and documentation time)* 5 times =360minute (6.83hours)

[b] nursing time/ patient = {[45min/ session + 30min (preparation time)/ session]*5 sessions/ 5 patients per group} + 8 minutes/ telephone follow-up * 3 times = 84minute (1.65 hour)

[c] values to be confirmed with the relevant parties (i.e. patient, caregiver and hospital administrative staff for venue rental)

eTable 2. Model parameters definition for cost effectiveness model

Parameters	Distribution type	Values
**Education**	Fixed proportions	
Probability of no hospital visits		0.86
Probability of non-A&E hospital visits		0.03
Probability of A&E hospital visits		0.10
**Empowerment**		
Probability of no hospital visits		0.88
Probability of non-A&E hospital visits		0.07
Probability of A&E hospital visits		0.05
**Education cost per person (USD)**	Fixed cost	553
**Empowerment cost per person (USD)**		1161
**Education**	Gamma(alpha, beta)	
Healthcare and societal costs for no hospital visits		5, 1407
Healthcare and societal costs for non-A&E hospital visits		4, 0.1
Healthcare and societal costs for A&E hospital visits		1, 0.1
**Empowerment**		
Healthcare and societal costs for no hospital visits		4, 154
Healthcare and societal costs for non-A&E hospital visits		2, 13766
Healthcare and societal costs for A&E hospital visits		4, 0.1
**Education**	Beta (alpha, beta)	
Utility (QALYs) for no hospital visits		37, 507
Utility (QALYs) for non-A&E hospital visits		59, 706
Utility (QALYs) for A&E hospital visits		4, 48
**Empowerment**		
Utility (QALYs) for no hospital visits		56, 521
Utility (QALYs) for non-A&E hospital visits		1, 9
Utility (QALYs) for A&E hospital visits		13,51
**Education**	Normal (mean, SD)	
Utility (SCHFI – symptom perception) for no hospital visits		0.2, 3.8
Utility (SCHFI – symptom perception) for non-A&E hospital visits		1.1, 3.3
Utility (SCHFI – symptom perception) for A&E hospital visits		2.6, 2.7
**Empowerment**		
Utility (SCHFI – symptom perception) for no hospital visits		1.6, 3.0
Utility (SCHFI – symptom perception) for non-A&E hospital visits		3.2, 3.4
Utility (SCHFI – symptom perception) for A&E hospital visits		1.8, 2.4
**Education**	Normal (mean, SD)	
Utility (SCHFI – management) for no hospital visits		-0.9, 4.2
Utility (SCHFI – management) for non-A&E hospital visits		-1.3, 7.8
Utility (SCHFI – management) for A&E hospital visits		-1.6, 3.3
**Empowerment**		
Utility (SCHFI – management) for no hospital visits		0.5, 4.3
Utility (SCHFI – management) for non-A&E hospital visits		-0.4, 2.8
Utility (SCHFI – management) for A&E hospital visits		2.0, 2.7

Note: CEA used 10,000 simulations and random seed number of 123. Utility distribution calculated as change from T0 to T2 follow-up. SCHFI utility calculated in change of units of minimally clinically important difference (≥7).

© 2022 Yu DS-f et al. *JAMA Network Open.*

eFigure 1: Acceptability Curve of Empowerment and education control for QALYS

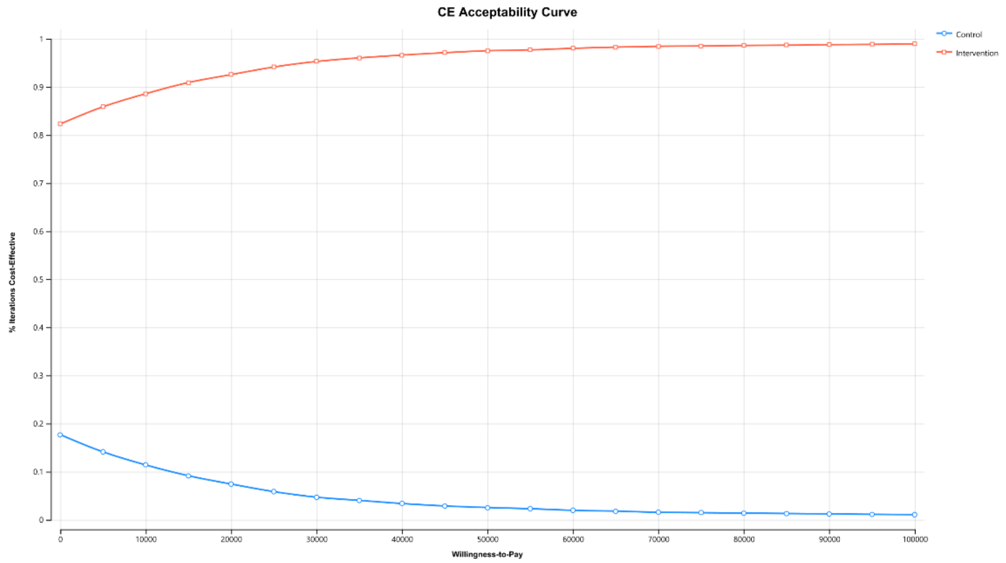

eFigure 2: Acceptability Curve of Empowerment and education control for SCHFI – Symptom perception

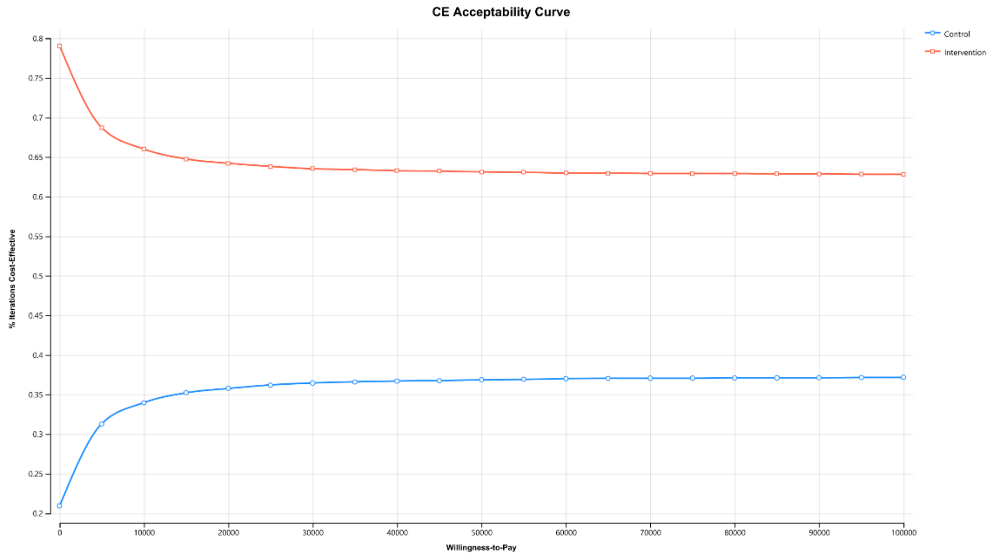

© 2022 Yu DS-f et al. *JAMA Network Open.*

eFigure 3: Acceptability Curve of Empowerment and education control for SCHFI – Management

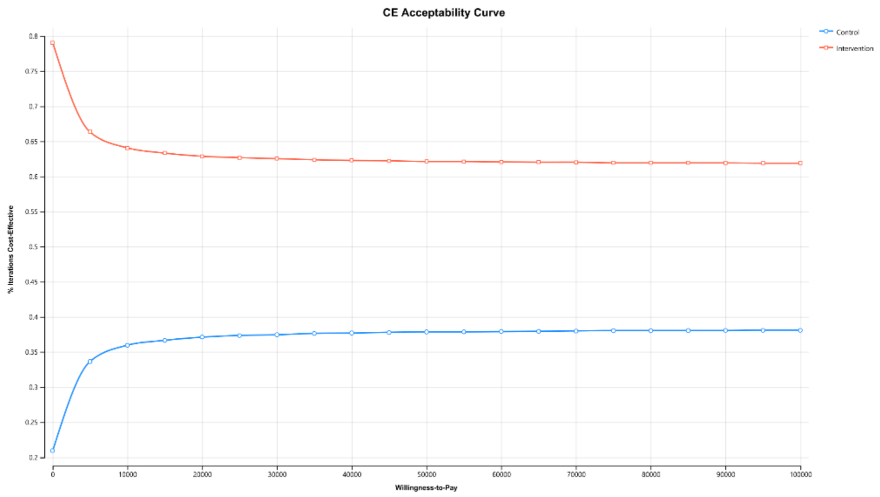

© 2022 Yu DS-f et al. *JAMA Network Open.*
