# Supplementary material for: Effectiveness and Cost-effectiveness of an Empowerment-Based Self-care Education Program on Health Outcomes Among Patients With Heart Failure: A Randomized Clinical Trial
Source: JAMA Netw Open. 2022 Apr 5;5(4):e225982. doi: 10.1001/jamanetworkopen.2022.5982 (PMC8984788; doi:10.1001/jamanetworkopen.2022.5982)
Supplement: Supplement 3. — Data Sharing Statement [file jamanetwopen-e225982-s003.pdf]

Data Sharing Statement

Yu. Effectiveness and Cost-effectiveness of an Empowerment-Based Self-care Education Program on Health Outcomes Among Patients With Heart Failure. *JAMA Netw Open*. Published April 05, 2022. doi:10.1001/jamanetworkopen.2022.5982

Data
Data available: No
